# Caretakers at the core: exploring worker perceptions of job satisfaction, training, cattlecare, and workplace community on feedyards in the United States

**DOI:** 10.1093/jas/skaf400

**Published:** 2025-11-20

**Authors:** Paxton A Sullivan, Libby Bigler, Mary Catie Cramer, I Noa Roman-Muniz, Lily N Edwards-Callaway

**Affiliations:** Department of Animal Sciences, Colorado State University, Fort Collins, CO 80523; Department of Animal Sciences, Colorado State University, Fort Collins, CO 80523; Department of Animal Sciences, Colorado State University, Fort Collins, CO 80523; Department of Animal Sciences, Colorado State University, Fort Collins, CO 80523; Department of Animal Sciences, Colorado State University, Fort Collins, CO 80523

**Keywords:** Beef Quality Assurance, caretaker well-being, cattle welfare, focus groups, social sustainability

## Abstract

The U.S. cattle feeding sector employs an important proportion of the agricultural workforce and is critical for meeting consumer expectations regarding how beef cattle are raised and cared for. Although extensive research has examined animal health, productivity, and performance in feedlots, the attitudes and perspectives of feedlot employees, who provide essential daily cattle care, have received limited attention. This study aimed to explore feedlot workers’ attitudes regarding job satisfaction, training, animal care, and workplace community using a mixed-methods approach. Feedlot workers (*n* = 329) from 29 feedlots nationwide participated in 95 focus group or interview discussions and completed a written survey. Survey responses indicated high levels of job satisfaction and pride in employees’ roles related to cattle care and welfare. Analysis revealed that employee agreement with key job satisfaction and cattle care statements was often associated with preferred language and age. Thematic analysis of focus group transcripts identified 13 primary themes: (1) Animal Health and Veterinary Care, (2) Stockmanship and Stewardship, (3) Continuous Improvement, (4) External Relations, (5) Safety, (6) Internal Communication, (7) Personal Backgrounds and Experiences, (8) Physical Environment, (9) Opportunities for Good Welfare and Positive Experiences, (10) Purpose and Role in Cattle Feeding, (11) Relationships, (12) Resources, and (13) Finance. Employees expressed a strong commitment to animal welfare and highlighted the importance of continuous improvement through ongoing training and hands-on learning opportunities. However, they also identified persistent challenges, including communication barriers, labor availability, equipment functionality, and extreme environmental conditions. Results from the logistic regression analysis of demographic factors suggest that language and age may influence employee experiences and perceptions within the feedyard environment. Addressing these challenges through targeted training improvements and supportive resources could significantly enhance both employee well-being and animal welfare. This research provides critical insights for incorporating workforce perspectives into comprehensive sustainability initiatives within the beef cattle industry.

## Teaser

This study explores the perspectives of feedlot workers on their roles, focusing on job satisfaction, training, animal welfare, and work community. It provides valuable insights into the challenges they face and highlights opportunities to improve both worker well-being and cattle care within the feedlot industry.

## Introduction

The U.S. beef cattle industry is complex involving many different sectors (i.e., cow-calf, stocker, and feedyard) that all contribute to raising cattle for beef. Beef production typically begins on cow-calf operations and continues through various segments (e.g., stocker, pre-conditioning, or backgrounding) before cattle are finished in feedyards. Feedyards are confined cattle feeding operations that promote animal growth and carcass quality. As of early 2025, 11.8 million cattle were on feed in U.S. feedlots (USDA NASS, 2025). Considering the number of cattle in feedyards across the U.S., the number of people employed in agriculture is relatively low; there were estimated to be approximately 267,000 livestock agriculture jobs in 2023, but this represented all livestock, not just feedyards (USDA ERS, 2023). The sheer scale of U.S. cattle feeding, combined with its labor demands and growing consumer expectations for how food animals are raised (Ritter et al., 2021), highlight the need for continued advancements in management, animal well-being, and sustainability of feedyard systems. While productivity and animal performance have been the focus of much research in this supply chain sector, the perspectives and needs of the human workforce (particularly those providing daily care) remain understudied (Calvo-Lorenzo, 2018).

Feedlot caretakers perform a wide range of job duties, from monitoring animal health and administering treatments to managing feed delivery and operating heavy equipment. These tasks are often performed under physically demanding conditions, variable and extreme weather, and irregular schedules. Additionally, many feedlot workers are required to make rapid, complex decisions, including deciding when euthanasia is necessary; euthanasia decision-making, a core component of livestock management, has been identified as a significant source of job-related stress among livestock caretakers (Wagner et al., 2020; Román-Muñiz et al., 2021). Additionally, feedlot workers frequently encounter occupational stressors related to workload, communication demands, and safety concerns (Ramos et al., 2018; Ramos et al., 2021a, 2021b). While previous research has largely focused on physical stress and safety risks (Ramos et al., 2021a; Carlo et al., 2023), less is known about how these workplace conditions intersect with attitudes toward animal care and perceptions of one’s role in supporting cattle welfare.

Evidence from other livestock sectors, including dairy and swine, indicates that caretaker well-being is closely tied to animal welfare outcomes, influencing both the quality of animal care and the overall sustainability of production systems ­(Daigle and Ridge, 2018; reviewed by Edwards-Callaway and Sullivan, 2024). Job satisfaction, adequate training, and a supportive workplace culture have been linked to improved animal handling and more consistent implementation of care protocols (Wilson et al., 2025). However, feedlot environments introduce unique structural and operational challenges, such as more frequent, intense, or time-constrained human–animal interactions (i.e., health checks, processing, loading for transport), that remain underrepresented in the literature. A recent study conducted by Sullivan et al. (2025) used focus groups to explore perceptions of job satisfaction, training, animal welfare, and workplace community among fed cattle transporters; transporters are not necessarily feedlot personnel, but they do interact directly with feedyards and are thus an affiliated group. Nearly all participants expressed pride in their work and acknowledged the importance of animal well-being, with high agreement on the effectiveness of current training programs. Still, transporters identified persistent barriers related to safety, communication, and facilities, underscoring the need for more cohesive support across supply chain segments and identifying areas where current training efforts could be improved (Sullivan et al., 2025). One of the only studies to explore feedlot caretaker perspectives found significant role-specific differences in perception, confidence, and workload among employees, highlighting critical gaps that may affect both cattle welfare and worker well-being (Ridge et al., 2019). While Ridge et al. (2019) reported that the Texas feedyard workforce is largely passionate about their job and are experienced in their job duties, many respondents indicated they were underpaid and overworked, emphasizing the need for improved support mechanisms and training within the feedyard sector.

The objective of this study was to explore the attitudes and perspectives of individuals responsible for the daily care of feedlot cattle, with the goal of understanding the intersection of human and cattle well-being on feedlots. Specifically, this research aimed to: (1) characterize caretaker perspectives and attitudes toward management practices related to cattle care on feedlots; (2) characterize their perspectives and attitudes regarding training, work environment, and work community; and (3) identify areas for improvement and inform the development of practical strategies, such as training and educational resources, to support both animal and caretaker well-being in feedlot systems.

## Materials and Methods

The study protocols were reviewed and approved by the Institutional Review Board at Colorado State University (CSU) prior to the project’s initiation (Protocol # 4419). The research was conducted between June and December 2023.

### Study population and recruitment

The population of interest for this study was feedlot caretakers, with an emphasis on those with direct cattle care responsibilities. However, during the course of the study the scope was expanded to include individuals that also participated indirectly in cattle care, for example, individuals who were responsible for maintaining cattle pens, feed bunks, and water tanks or individuals that participated in weighing and processing but not direct care. This expansion made the study more robust as it included the entire team that was responsible for cattle care on feedyards. Feedlots were recruited by co-authors of this manuscript either directly or through contacts within the beef industry. Feedlots of any size and location were eligible to participate, provided they had cattle on feed. Once a feedlot agreed to participate in the study, a date to visit the operation to conduct the focus groups was scheduled. Participants represented employees from 11 states spanning the Western (i.e., California, Colorado, Idaho, Washington; *n* = 9), Midwestern (i.e., Iowa, Kansas, Nebraska; *n* = 12), Southwestern (i.e., Oklahoma, Texas; *n* = 5), and Southeastern (i.e., Alabama, North Carolina; *n* = 3) regions of the United States.

### Focus group and survey delivery and format

Focus group and interview discussions were facilitated by research team members with employees from participating feedlots. Sessions typically occurred during the workday, often aligning with lunch breaks; however, the scheduling was flexible and ultimately determined by the preferences of the feedlots. In some cases, multiple smaller focus groups were conducted at a single feedlot to accommodate their preferences and workflows. The majority of sessions were conducted as focus groups with more than two participants; however, a small number (<10) were conducted with one or two individuals. Participants could choose to participate in Spanish or English focus groups based on their language preference, with Spanish-language discussions led by a fully bilingual native Spanish speaker; Spanish-speaking facilitators were not consistent across focus groups but all of them had the same level of proficiency.

To foster a relaxed and comfortable setting, breakfast or lunch was provided, and discussions were held in private rooms with only the facilitator and participants present. Employees and supervisor or management-level participants attended separate sessions to ensure open and candid communication and to minimize concerns about power dynamics.

Before the focus group began, the facilitator explained the research objectives and obtained verbal informed consent from all participants. Participants were informed that participation was voluntary, confidential, and that they could leave the focus group at any time. Participants also completed a brief written survey before the discussion, which included six Likert-scale questions addressing topics including training, job satisfaction, cattle well-being, and workplace community and environment, along with six demographic questions. Surveys were delivered on paper and provided in Spanish or English. The individual that was facilitating the focus group also facilitated the survey administration. Participants that were unable to read the written survey were guided verbally through the survey and either marked the answers themselves with guidance on what the options were or, if they preferred, the facilitator would fill in the survey for them.

Once surveys were completed, the focus group discussion began, guided by a structured set of 22 questions. These questions were developed by the research team in addition to colleagues and other industry stakeholders with extensive experience working with feedyard employees. Once drafted, the questions were shared with a small group of collaborators to evaluate clarity and validity. These questions were divided into three main categories: (1) job satisfaction, (2) training, cattle care, and animal well-being, and (3) work community and environment. A detailed question list can be found in Table 1. The facilitators were trained to only ask the predetermined set of questions and not add opinion to the discussion; follow up questions to ask for more detail by the facilitator were permitted. Discussions were audio recorded using the Voice Memos app on an iPad (Apple Inc., Cupertino, CA, USA). At the conclusion of each session, participants received a $20 cash incentive to thank them for their time and contributions. Although thematic saturation was achieved prior to the completion of all interviews and focus groups, focus group sampling continued to ensure inclusion of participants from feedyards across a range of geographic regions and operation sizes.

**Table 1.** Focus group questions for feedlot focus groups

Question (Q)	Sub-question
***Related to job satisfaction***	
**Q1: What is your favorite part of your job? **	
**Q2: What is your least favorite part of your job? **	
**Q3: What is one thing that would make your job better or easier? **	
**Q4: What makes you proud of doing your job? **	
**Q5: Do you see yourself continuing to work in the cattle feeding industry? **	
***Related to training, cattle care, and animal well-being***	
**Q6: What did you know about taking care of cattle before starting your current job? **	
**Q7: What have you learned about taking care of cattle since being at your current job? **	
**Q8a: What kind of training have you received to do your job? **	Q8b: How has your training prepared you for taking care of cattle?
	Q8c: Is there more training that would be helpful to you to perform your job?
**Q9a: What does cattle well-being mean to you? Define it in your own words. **	Q9b: Is that different for other animals, such as pets or horses?
**Q10: What is the most critical component of taking care of cattle?**	
**Q11a: What cattle well-being challenges do you experience on the feedyard? **	Q11b: How do these challenges make you feel?
***Related to work community and environment***	
**Q12: What do you like about your current work environment? **	
**Q13a: How would you describe your work community? **	Q13b: Do you spend time together inside or outside of work? Do you want more opportunities to do so?
**Q14a: In general, how do you communicate with your coworkers (e.g., call them on the phone, text them, talk to them in person)? **	Q14b: More specifically, how do you discuss problems about taking care of cattle with your coworkers?
**Q15: Do you feel comfortable asking for help when solving a problem related to taking care of cattle at work? **	
**Q16: Is there anything else you would like to share? **	

### Statistical analysis

#### Quantitative analysis

Responses from the written surveys were collected and organized in a Microsoft Excel spreadsheet (Microsoft Corporation, Washington, DC, USA). Descriptive statistics were used to summarize both the Likert-scale data and demographic information.

For regression, Likert responses were collapsed to binary outcomes: Strongly Agree/Agree coded as 1 (Agree) and Strongly Disagree/Disagree coded as 0 (Disagree). The Strongly categories were rare across questions, so adjacent categories were collapsed to avoid sparse cells and unstable estimates. Each question (Q1–Q6) was modeled separately using single-level logistic regression with a logit link.

Although respondents were nested within feedlots, a feedlot-level random intercept was not retained. In preliminary mixed-effects logistic models (lme4::glmer), the between-feedlot variance component was estimated at (or near) zero and models were singular, indicating negligible clustering relative to within-feedlot variability. Likelihood-ratio tests comparing models with versus without the random intercept showed no improvement in fit, and fixed-effect estimates were materially unchanged. Given minimal between-feedlot variability and small/uneven counts per feedlot, single-level models are reported for parsimony and stability.

Predictors in all models were respondent language (Spanish vs. English [referent]) and age in years (continuous variable). Because language, ethnicity, and country of origin were highly collinear, only language was retained in each model. Similarly, age and years of experience were strongly correlated; only age was included to avoid redundancy. Observations with missing data on any model variable were excluded, so analytic *n* varied by question. Models were estimated by maximum likelihood and summarized as odds ratios (ORs) with 95% Wald confidence intervals (Cis); two-sided α = 0.05 defined statistical significance. Predictor contributions were evaluated with likelihood-ratio χ^2^ tests from analyses of deviance. All analyses were conducted using R version 4.4.2 (R Core Team, 2024).

#### Qualitative analysis

Audio recordings from each focus group were transcribed using an online transcription platform (Auris AI; Singapore). Transcripts were then reviewed for accuracy by members of the research team through a multi-step verification process. This included listening to the original audio, correcting any errors generated by the AI transcription, anonymizing the data by removing identifying information, and reformatting the text so that each speaker (whether facilitator or participant) was clearly delineated on a separate line. Spanish-language transcripts followed the same process, with transcription verification and translation conducted by a fully bilingual, native Spanish speaker to ensure cultural and linguistic accuracy.

A critical realist approach guided the analysis, acknowledging that while there is an external reality, the understanding of it is partial and socially situated. This orientation, combined with an inductive thematic analytical approach (Braun and Clarke, 2006), allowed for the systematic identification and description of patterns and themes from the data without being constrained by pre-existing theories. This approach enabled a rigorous and structured exploration of participant experiences while maintaining a commitment to representing the complexity and nuanced reality of their accounts.

Nine researchers independently reviewed and familiarized themselves with all transcripts before generating initial codes. From these codes, the research team collaboratively identified patterns and developed a set of preliminary themes. A comprehensive codebook was then constructed, including definitions and illustrative examples for each theme. To establish consistency, seven researchers were chosen to code and conducted intercoder reliability testing using one transcript. Discrepancies were resolved through group discussion, and the codebook was revised accordingly. Additional rounds of intercoder reliability were conducted with a new transcript each time, until agreement among coders was reached. Once finalized, the codebook was used to analyze the remaining transcripts. These were deidentified and randomly assigned to coders to ensure an even distribution of workload. Each coder independently applied the final codes to their assigned transcripts. The use of intercoder reliability was employed not to claim a single, objective truth, but to enhance the dependability and consistency of the coding process, ensuring that the identified themes were applied systematically across the dataset. Intercoder reliability was assessed through percentage agreement (≥80%) rather than Cohen’s kappa, as the latter can be misleading in qualitative thematic coding where codes often overlap and vary in prevalence; discrepancies were resolved through iterative discussion to ensure consistency and dependability.

The first coder earned a Bachelor’s and Master’s degree in Animal Science and is currently pursuing a Ph.D. in the same field, with research centered on human and animal well-being within the beef supply chain. The second coder is a faculty member at the university, whose research focuses on food animal welfare and who also brings previous experience from multiple sectors of the beef industry. The third coder completed her Bachelor’s degree in Dairy Science, worked in the dairy industry, and now serves as a research associate at the university. The fourth coder holds a Bachelor’s degree in Animal Science and is enrolled in a combined Master’s/DVM program, studying fed cattle well-being. The fifth and sixth coders are fourth-year Animal Science undergraduate students who actively participate in research within the behavior and welfare laboratory. Finally, the seventh coder is a second-year Master’s student studying livestock behavior and welfare; prior to beginning her graduate studies, she taught high school agriculture education.

## Results

### Quantitative results

#### Demographics

Three hundred twenty-nine individuals (*N* = 329) from 29 feedyards across the United States were included in the study. The participating feedlots represented a wide range of one-time cattle capacities: four had fewer than 5,000 head, nine had between 5,000 and 9,999 head, five had between 10,000 and 19,999 head, six had between 20,000 and 49,999 head, and five had greater than 50,000 head. Sample population demographics are described in Table 2. Most respondents surveyed were men (86.3%, *n *= 283), followed by a smaller proportion of women (13.7%, *n *= 45). A wide distribution of age groups was represented in the study, with survey respondents ranging in age from 18 to 65 and over; the greatest number of respondents reported being within the age ranges of 25 to 34 (26.8%, *n *= 85), 45 to 54 (21.1%, *n *= 67), and 35 to 44 (18.0%, *n *= 57). Smaller proportions of respondents were between the ages of 18 to 24 (12.6%, *n *= 40), 55 to 64 (15.5%, *n *= 49), or 65 and older (6.0%, *n *= 19).

**Table 2.** Feedyard demographics (*n* = 329) collected from the written ­survey

Variable	*n*	Frequency (%)
**Language (*n* = 329)**		
** English**	177	53.8
** Spanish**	135	41.0
** 2 languages^1^**	17	5.2
**Ethnicity (*n* = 324)**		
** Hispanic or Latino**	173	53.4
** Non-Hispanic or Latino**	151	46.6
**Gender (*n* = 328)**		
** Man**	283	86.3
** Woman**	45	13.7
**Age (*n* = 317)**		
** 18–24**	40	12.6
** 25–34**	85	26.8
** 35–44**	57	18.0
** 45–54**	67	21.1
** 55–64**	49	15.5
** 65+**	19	6.0
**Region of origin^2^ (*n* = 329)**		
** Africa**	6	1.8
** Central America**	17	5.2
** Mexico**	136	41.3
** South America**	2	0.6
** United States**	168	51.1
** No response**		
**Length of time working (*n* = 327)**		
** Less than 6 mo**	35	10.7
** 6 mo to 1 yr**	27	8.3
** 1 to 2 yr**	53	16.2
** More than 2 yr, but less than 10 yr**	131	40.1
** 10 yr or more**	81	24.8

1 Respondents checked both Spanish and English (*n* = 10) or English and Portuguese (*n* = 1) as their preferred languages.

2 Other regions were offered as choices but were excluded from the table because they were not selected by any respondents.

Just over half of respondents identified as Hispanic or Latino (53.4%, *n *= 173), with 46.6% (*n *= 151) identifying as non-Hispanic or Latino. Most respondents indicated that English was their preferred language (53.8%, *n *= 177), followed by 41.0% (*n *= 135) who preferred Spanish, and 5.2% (*n *= 17) who preferred to speak both English and Spanish.

Most participants worked in non-supervisory roles (74.8%, *n *= 246), while 25.2% (*n *= 83) held supervisory positions. Respondents reported a range of experience working in their current feedyard, with the greatest proportion reporting more than 2 yr but less than 10 yr of experience (40.1%, *n *= 131), followed by 10 yr or more (24.8%, *n *= 81) and 1 to 2 yr (16.2%, *n *= 53). Smaller proportions reported having worked in their current role for less than 6 mo (10.7%, *n *= 35) or between 6 mo and 1 yr (8.3%, *n *= 27). Finally, respondents originated from multiple regions, yet the largest proportion reported being from the United States (51.1%, *n *= 168), followed by Mexico (41.3%, *n *= 136).

#### Likert-scale questions and regression analysis of agreement statements

Participant responses to statements related to job satisfaction, cattle care, training, and the work environment were generally positive, with most respondents expressing agreement across all the statements (Table 3). Nearly all participants indicated that they either “Agreed” or “Strongly Agreed” with the statement “I have pride in the job I do” (29.7%, *n* = 95; 66.6%, *n* = 213, respectively).

**Table 3.** Feedyard agreement with Likert scale questions (*n* = 328)

Statement	*n* total	Agreement	*n*	Frequency (%)
**“I have pride in the job I do.”**	320	Strongly Agree	213	66.6
		Agree	95	29.7
		Disagree	5	1.6
		Strongly Disagree	7	2.2
**“The training I have received at this job has prepared me well for taking care of cattle as part of my job duties.”**	328	Strongly Agree	164	50.0
		Agree	151	46.0
		Disagree	8	2.4
		Strongly Disagree	5	1.5
**“Animal well-being is a critical component of my job.”**	326	Strongly Agree	231	70.9
		Agree	84	25.8
		Disagree	7	2.1
		Strongly Disagree	4	1.2
**“I have difficulty communicating with my coworkers or supervisors.”**	326	Strongly Agree	29	8.9
		Agree	58	17.8
		Disagree	138	42.3
		Strongly Disagree	101	31.0
**“I feel like I am a valued member of my team.”**	328	Strongly Agree	145	44.2
		Agree	164	50.0
		Disagree	13	4.0
		Strongly Disagree	6	1.8
**“I am satisfied with my current role and responsibilities at this job.”**	328	Strongly Agree	166	50.6
		Agree	143	43.6
		Disagree	15	4.6
		Strongly Disagree	4	1.2

The majority of respondents reported being satisfied with their current job responsibilities, with most selecting “Agree” or “Strongly Agree” in response to the statement “I am satisfied with my current role and responsibilities at this job” (43.6%, *n* = 143; 50.6%, *n* = 166, respectively).

In response to the statement “Animal well-being is a critical component of my job,” most participants expressed agreement, with 25.8% (*n* = 84) selecting “Agree” and 70.9% (*n* = 231) selecting “Strongly Agree.”

Responses to the training-related statement, “The training I have received at this job has prepared me well for taking care of cattle as part of my job duties,” were also favorable, with 46.0% (*n* = 151) of respondents indicating “Agree” and 50.0% (*n* = 164) indicating “Strongly Agree.”

Responses to the communication-related statement, “I have difficulty communicating with my coworkers or supervisors,” were more variable; while the majority of respondents either “Disagreed” or “Strongly Disagreed” with the statement (42.3%, *n* = 138; 31.0%, *n* = 101, respectively), a smaller proportion selected “Agree” or “Strongly Agree,” (17.8%, *n* = 58; 8.9%, *n* = 29, respectively).

Lastly, 50.0% of respondents “Agreed” (*n* = 164) and 44.2% “Strongly Agreed” (*n* = 145) with the statement, “I feel like I am a valued member of my team.”

Single-level logistic regression models were fit for each of the six survey questions, with predictors for respondent language (Spanish vs. English [referent]) and age in years. Odds ratios (OR) and 95% confidence intervals (CI) are presented in Table 4.

**Table 4.** Logistic regression of language and age predictors of feedlot caretaker attitudes and job-related perceptions

*Predictor*	*Odds ratio*	*CI*	*P-value*
Q1. I have pride in the job I do.			
**(Intercept) **	64.84	8.91-711.53	<0.001
***Preferred Language***			
**English **	*Referent*	–	–
**Spanish **	0.25	0.05–0.91	0.048
***Age in years***	1.00	0.95–1.04	0.879

Q2. The training I have received on this feedyard has prepared me well for taking care of cattle as part of my job duties.			
*Predictor*	*Odds ratio*	*CI*	*P-value*
**(Intercept) **	46.50	7.63-395.48	<0.001
***Preferred language***			
**English **	*Referent*	–	–
**Spanish **	1.31	0.38-5.12	0.675
***Age in years***	0.98	0.94-1.03	0.455

Q3. Animal well-being is a critical component of my job.			
*Predictor*	*Odds Ratio*	*CI*	*P-value*
**(Intercept) **	594.19	52.80-14435.14	<0.001
***Preferred language***			
**English **	*Referent*	–	–
**Spanish **	0.74	0.20–2.76	0.645
***Age in years***	0.94	0.89–0.99	0.014

Q4. I have difficulty communicating with my coworkers or supervisors.			
*Predictor*	*Odds ratio*	*CI*	*P-value*
**(Intercept) **	0.22	0.09-0.51	<0.001
***Preferred language***			
**English **	*Referent*	–	–
**Spanish **	2.04	1.20-3.50	0.008
***Age in years***	1.00	0.98-1.02	0.774

Q5. I feel like I am a valued member of the feedyard team.			
*Predictor*	*Odds ratio*	*CI*	*P-value*
**(Intercept) **	29.04	5.72–186.69	<0.001
***Preferred language***			
**English **	*Referent*	–	–
**Spanish **	0.30	0.09–0.85	0.030
***Age in years***	1.00	0.97–1.04	0.836

Q6. I am satisfied with my current role and responsibilities at this feedyard.			
*Predictor*	*Odds ratio*	*CI*	*P-value*
**(Intercept) **	6.86	1.70–30.41	0.008
***Preferred language***			
**English **	*Referent*	–	–
**Spanish **	1.01	0.38–2.86	0.977
***Age in years***	1.02	0.99–1.06	0.259

For Q1 (pride in one’s job), Spanish-speaking respondents had significantly lower odds of reporting pride in their work compared with English-speaking respondents (OR = 0.25, 95% CI: 0.05–0.91, *P* = 0.048). Age was not associated with job pride (p = 0.879). For Q2 (training preparedness for cattle care), neither language (OR = 1.31, 95% CI: 0.38–5.12, *P* = 0.675) nor age (*P* = 0.455) significantly predicted agreement. For Q3 (importance of animal well-being in one’s job), younger respondents were more likely to agree that animal well-being was a critical job component, with odds of agreement decreasing with each additional year of age (OR = 0.94, 95% CI: 0.89–0.99, *P* = 0.014). No significant differences were observed by language (*P* = 0.645). For Q4 (difficulty communicating with coworkers or supervisors), Spanish-speaking respondents were significantly more likely to report difficulty compared with English-speaking respondents (OR = 2.04, 95% CI: 1.20–3.50, *P* = 0.008). Age was not associated with reported communication challenges (*P* = 0.774). For Q5 (feeling valued as a member of the feedyard team), Spanish-speaking respondents had significantly lower odds of agreement compared with English-speaking respondents (OR = 0.30, 95% CI: 0.09–0.85, *P* = 0.030). Age was not associated with this outcome (*P* = 0.836). For Q6 (satisfaction with current role and responsibilities), no significant effects were observed for either language (*P* = 0.977) or age (*P* = 0.259).

### Qualitative results

Ninety-five focus groups were conducted (*N* = 95). Thematic analysis of the focus group transcripts identified 13 themes, which were: (1) Animal Health and Veterinary Care; (2) Stockmanship and Stewardship; (3) Continuous Improvement; (4) External Relations; (5) Human and Animal Safety; (6) Internal Communication—with subthemes of: (6a) Methods and Preferences, (6 b) Successes, and (6c) Challenges; (7) Personal Backgrounds and Experiences; (8) Physical Environment; (9) Providing Opportunities for Good Welfare and Positive Experiences; (10) Purpose and Role in Cattle Feeding Sector; (11) Relationships; (12) Resources; and (13) Finance. These themes, as well as their associated definitions and excerpts from the transcripts exemplifying each theme are shown in Table 5. The final number of themes was determined by the point of thematic saturation, as no new or significant themes were identified from the data after this point. While this is a relatively large number, each theme was deemed distinct and essential for a comprehensive representation of the findings.

**Table 5.** Feedlot focus group theme definitions, main concepts within each theme, and a sample of quotations from responses to all focus group questions (*n* = 95)

Code name		Definition/Examples
**Animal Health and Vet Care**	Related to animal health and veterinary care—specific to identifying and/or treating sick animals, administering medicines or other vet care; comments about performance or productivity related to animal health; meeting withdrawal times or talking specifically about specific regulations for animal health products.	“*Well, as I told you, diseases are different. Every disease or every animal is sometimes different. Eh, there are animals that bring temperature. When you grab them, they don’t bring temperature, but simply if you see the animal’s countenance, the face, the eyes, the glassy vision, the very drooping eyes, then that’s where you have to say, I give, I give you medicine or I don’t. But that’s where I’m going to take a risk because if you don’t give it the medicine, the calf is going to get worse.*” “*…when I got to the feed yard it was different because the way the vaccine was, the vaccine protocols was way different than cow-calf operations.*” “*Especially like if I’m doctoring an animal and like on our program it’ll say they have 21 days till they ship. But if I’ve been told they’re shipping next week and I don’t remember. Like we have a lot of cattle going out in and out this week. And I’ll be like I don’t remember when they’re going out and since it’s so short I don’t want to get stuck with a withdrawal.*”
**Stockmanship and Stewardship**	Broad category related to comments around cattle stockmanship (i.e., animal handling, animal behavior, working with cattle, stress, keep cattle calm, etc.); regarding any feelings toward the cattle in their care or general care overall; feelings of respect toward cattle or honoring the animal; human-animal bonds and/or interactions; any mention of adhering to animal care guidelines, standards, or best practices (following BQA for animal care procedures/standards, all-natural program, etc.); liking to work with animals (including cattle).	Animal handling/Behavior/Stress: “*The whole, just even that cattle handling, you know, where, you know, before when I was a kid, everything was a square box and everybody got ran over and it was a really ugly thing. Nobody wanted to work calves because you just take a whip and every time, you know, nowadays, you know, Temple Grandin, you know, after 20 years of it, mind you, now we all know what a sweep tub is. We all know what an easy corner is. Now we know the idea that, you know, fast is slow, but slow is smooth and smooth is fast. You know, we’ve all been, we’ve actually all probably grown up with that now.*” “*And then you have that final day loading them out, just making it as least stressful as possible, not yipping and hollering and beating and all that stuff.*” Feelings toward cattle: “*It stresses me out and it’s kind of depressing.*” “*And then on the euthanasia one, that one’s usually harder for me because I know what they’re thinking. And it’s like, hey, I need you to go put this one down. And they’re like, okay. So, I hate to ask them, you know, it’s just because they don’t, they don’t, you’ll meet…my cattle manager, and he is the kindest person you’ll ever see. Like if the cattle are not comfortable, it, like, it hurts him. And so, to go tell him, put one down, it’s like, he’s having to quit, and he doesn’t want to quit. And I don’t like to hurt his feelings because he’s a nice guy.*” Following best practices: “*And so, and then after I came back, you know, implementing a lot of those things into our workforce and stressing the importance of you know, just standard BQA guidelines and, you know, changing how we did things.*”
**Continuous Improvement**	Related to any mention of learning or training, getting feedback from assessments/audits/outside consultants. Can be about the method, type, or delivery of training, the relevancy of training, limitations of training, how people learn best (e.g., hands-on, on-the-job), any mention of BQA trainings specifically or similar program, learning from others, learning something new every day, experience as a teacher. Comments can also be about getting help, advice, or training from outside consultants (e.g., veterinarians, nutritionists, universities, etc.).	“*We work with the PAC group, the veterinary group, and they taught me about everything I know. Just how to position yourself and what to watch for when you’re walking through the pens.*” “*We consult our vets. We consult our nutritionists. You know, there’s a world of experts out there, and you might want to take advantage of that. There’s all of them. And you have to kind of pick and choose a little bit what fits in your operation, but there’s a lot of expertise out there that’s free for the taking, basically.*” “*I really like and would like to see a continue or more of just general training that’s offered to producers. And whether that’s through the stockmanship and stewardship. The CCA, CLA, all our different organizations, our stock growers association, the BQA, you know, things like that. I’ve done a lot of Zoom meetings. It’s very helpful for me who’s never had formal training or anything like that.*”
**External Relations**	Comments regarding interactions, relations, or communication with anyone outside of the feedlot or feedlot company; comments can be about customers, the public, or other areas of the supply chain (e.g., the ranch, the packer, other feedlots); comments regarding managing perceptions and expectations of anyone external to the feedlot.	“*Well, the public is always… The public, I don’t know how you could educate them. Most of the time it’s brainwashed.*” “*We’ve had some customers who buy the beef because we do a lot of exporting form Japan. It used to be every couple of years, the same gentleman, he works for [company name] and he’s over in Japan and he would bring some businessmen in. I’m sorry. They were very nice people, but in suits.*” “*I get to do some of the stuff here in the office, work with a little bit more with the customers. So, like, the therapies, the customer requested it, and I said, let’s do it. Because I wanted to see how it worked, in all honesty.*”
**Human and Animal Safety**	Related to any comments to do with animal or human safety, or anything that may impact animal or human and safety specifically. For animals, comments may cover sentiments about keep cattle safe from harsh weather or bad pen conditions, or keeping them from getting injured during handling—these are just a few examples; animal safety could be a very broad topic. For humans, these comments may refer to the inherent dangers of the job, getting injured on the job, the physical toll this job has on the body, staying safe during animal handling, etc.	“*We’re in an unsafe situation all the time, you know. I mean, we have a—I know we’ve all been beat with the statistics at every one of our safety meetings, but, man, we’re all lucky to be alive.*” “*The heat. From cold to hot it’s very dangerous for animals and everything. They are up against time.*” “*We’ve learned a lot about the safety of, of the cattle. The handling also for the same. The respect towards the cattle as well, through the meetings that are given to us about safety and all that.*”
**Internal Communication**	Related to any communication with people within the feedlot or feedlot company (e.g., other employees, supervisors, managers), this theme does not include people in other areas of the supply chain; comments can broadly be about the method of communication used or about the preference for the method of communication. Any comments referring to communication successes (e.g., comfort asking for help, efficiency, having a supportive team) or communication challenges or failures (e.g., breakdown between departments, language barriers, generational differences, etc.) fall under this theme.	Methods and preference of communication: “*I mean, I send out a group text all the time, every day which is tough. I know the younger kids are good with phones and all that stuff, and then I got a few older guys that maybe prefer, you know face to face or whatever.*” “*You know cowboys are all in here in the morning so then if we got specific issues, I just talk to them about it and tell them to keep an eye on this or that. And then yeah if there’s a problem I see, if it’s on the feed or equipment side just get al. the crew together on that crew and say this is what I’m seeing and all that.*” Successes: “*…other than that I think everybody here is usually pretty good about just talking through it and moving on and getting it fixed.*” “*And everybody’s nice. You can, you have a question, you can pretty much go to anybody and ask them and they’re not going to jump, you know, jump your back or anything.*” Challenges: “*There’s way too many channels and bosses and all this stuff to go through to get your issue taken care of.*” “*For me, what I would like most was for there to be communication throughout the whole work team. Communicate with each other in order to know everything we are going to do. Because sometimes they say one thing to some people and another to others, and then we don’t even know what’s next. So, if there is no communication between us, we will never agree on anything.*”
**Personal Backgrounds and Experiences**	Related to any comments regarding the respondents’ personal identity, beliefs, religion, culture, gender, race/ethnicity/nationality, and other backgrounds and experiences that have shaped who they are today; comments may also refer to specific successes or challenges due to the respondent’s specific background, experience, or identity (i.e., being bilingual and able to communicate with lots of people; or being a woman in ag.) Comments referring to the specific personal characteristics and/or skills necessary to do the job; why the workers are good at what they do.	“*Well, most of us come from a home ranch. Not all. I don’t want to put that out there. A lot of us come from a youth of being kind of raised up in a ranch environment. We were raised by family, maybe not even our close family, but extended family as well that brought us into the trade, you know, and you start to learn about the horses and the cows and what it takes to make a fat cow.*” “*…since I was a child I always dreamt of being a cowboy, since I was a child that was my dream like that. My biggest dream in life was to be a cowboy. I watched movies on the television, I, I want to be a cowboy, I want to herd cattle. And then you can imagine, well I was born in Mexico, and I had to come here and, and to arrive and find a job where its as if, I feel as if I was paid for, for fulfilling my dream.*” “*What makes me feel proud because here I arrived and learned many things that I didn’t know. Where I come from I barely rode a bicycle. And here I feel proud that I learned to operate machinery, heavy equipment and many things that I had not learned here I came to learn.*”
**Physical Environment**	Comments pertaining to the weather or environment; comments may be specific to the type of conditions or elements (e.g., rain, snow, dust, mud, heat, ice, etc.) and challenges associated with these conditions (e.g., riding pens in bad pen conditions). Preferences or dislikes about the physical environment (inside/office, vs outside/weather).	“*500 times easier if I could control the weather.*” “*…70 degrees during the day and 45 degrees at night and cattle, cattle love that environment, but we don’t live in that…*” “*Riding through two foot of mud in the pen.*”
**Providing Opportunities for Good Welfare and Positive Experiences**	A broad category of comments focused on providing cattle with positive experiences and opportunities for good welfare. Comments may refer to cattle comfort, providing basic needs such as water and feed, giving animals a clean and ample space to lay and perform natural behaviors	“*I think, animal welfare is animal welfare. You take, whether it’s—whether you’re taking care of your pet Parakeet, or you have 10,000 head of cattle. You want, you want to be taken care of in a, you know, humane situation. You don’t…want to starve, you don’t want to be over fed, you—in the environment to put it in you want it taken care of you know.*” “*Making sure they’re looked after. Making sure they’ve got plenty to eat. Their nutrition is right. It’s just because if you don’t look after them, they’re not going to look after you.*” “*Taking them to the pen and going to a clean, clean environment with clean water, and you know we did fresh bedding and kind of just the little things you got to make sure you’re doing right.*”
**Purpose and Role in Cattle Feeding Sector**	Sentiments from people about their roles and responsibilities taking care of/feeding cattle; satisfaction in problem solving and executing plans successfully; sense of accomplishment in a job well done; their pride in their job; pride in executing tasks/responsibilities/specific job duties; significance of their contribution to a team and the larger industry; feeding people and the world; seeing cattle progress through the finishing phase. Comments referring to why feedlot employees do what they do.	“*…seeing the cattle come in as little guys, and a little bit rough, and then watch them go out finished and look fat and healthy.*” “*Well, the proudest thing for me is, um, riding the horse, moving the cattle, and seeing the results at the end when the cattle leave the pens for sale. It’s what makes me feel the proudest because I know I’m doing a job, and not only that, but a meal is going to homes for families.*” “*I work in the yard, my job is to manage the machine, to maintain this, as now that the rain is coming we have to make the space for the cattle and another space, another road for the water tank, it is a round job, all year round and I love it, I have been working here in this company for 23 years and that is my job. I have been doing it all my life.*”
**Relationships**	Related to any interactions with people within the feedlot or feedlot company and does not include people in other areas of the supply chain; comments related to relationships with coworkers, and specific words to describe the environment, such as family, dysfunctional, brotherhood, teamwork, etc.; comments related to the community of people at work, time spent together.	“*Oh, we do spend time together. Some more than others, you know, we have a diverse group here, but I, I enjoy spending time with them.*” “*And for the coexistence here with friends, its nice, and everything. As in everything! As in every family sometimes there is, always…a day that we arrive and we fight and oh this and that. But in the end we are family and here we are and we all unite because I believe the same, the work for, for what we do.*” “*Mine would be the people. That’s, I enjoy working with—they’re considered family to me out here. So I enjoy working with the people.*”
**Resources**	Comments related to resources for feedlot personnel to do their jobs—these resources may include facilities (space, infrastructure, etc.), technologies, equipment, and records (record keeping, data management, etc.); comments may also refer to labor—to do with the changing workforce, availability, quality of labor, attitudes, work ethics—and time (or lack thereof).	“*We can watch the feed being fed now through our phones as it comes off. You know the technology up here is just from when I started where this now is just absolutely phenomenal.*” “*For me, the availability of employees that are knowledgeable is not very good. I mean, it seems like they’re getting fewer and fewer and harder to find.*” “*New equipment…. more equipment, more operators…*”
**Finance**	Comments referring to profit/loss, making or losing money, paychecks, production losses or gains, or any other comments associated with some financial component(s).	“*So I think we do a better job of, than anybody of taking care because it affects the profit and loss.*” “*Because if you’re there to make money, you’d be a plumber or electrician, you wouldn’t be an agricultural worker.*” “*And then we have, yeah, and then there’s a little bit of, I would say, discontent with like, even the beef board, the way they get their money taken a dollar from everybody. Are they doing as good a job as they could with that money? I don’t know.*”

Results are presented by question blocks which focused on (1) job satisfaction, (2) training, cattle care, and animal well-being, and (3) work environment and community. The quotes selected for this analysis aim to highlight key ideas and connections across themes, rather than simply reporting the frequency with which they occurred.

### Related to job satisfaction

Participants in this section responded to five questions regarding job satisfaction (Table 1). These questions focused on identifying the most and least enjoyable parts of their job, potential changes to improve or simplify their work, sources of pride in their role, and their outlook on a future within the beef industry. Across these discussions, all themes were represented to varying degrees, highlighting the wide range of factors influencing both satisfaction and dissatisfaction among feedlot workers.

#### Positive attributes and job-related successes

Respondents frequently highlighted Stockmanship and Stewardship, Purpose and Role in Job, and Relationships as central to their job satisfaction. When discussing Stockmanship and Stewardship, participants frequently spoke of animal handling, human-animal bonds, and adherence to animal care guidelines. For instance, one participant reflected, “*I enjoy being able to take care of the animals…. I just, I really like being able to take as good care as I can of them before they go elsewhere.*” Another participant shared, “*Definitely working with animals, working with cattle. Most of us it’s a lifelong passion. And some of us are just getting into it and it’s growing on them. But I’m here for the cattle.*”

Similarly, the Purpose and Role in Job theme was present in discussions about personal roles and responsibilities. Respondents expressed pride and satisfaction in their work, sharing sentiments like, “*Always learning, no matter how long we’ve been here, there’s always something new, something to change. There’s always something, a way to make things better. And all of us here strive to make things better*.” One participant summarized this sense of responsibility by saying, “*…every day we got goals and work to do. It’s a high responsibility.*” This sense of responsibility often intersected with other themes, as seen in the following quote: “*The whole thing, people, cattle, the environment, the challenge of it, and just, just the industry itself.*” A focus group participant further explored their favorite part of the job by connecting the Stockmanship and Stewardship and Purpose and Role in Job themes: “*Part of my job is quality control, so I get to go out and watch processes and whatnot. Just seeing the cattle. I know it sounds cheesy, but just being there and feeling more connected to what we do as a whole. And connecting data to our product and getting to see the animals. That’s probably my favorite part.*”

The Relationships theme was prominent, with many respondents sharing that the interactions with their coworkers were among their favorite parts of the job. Comments such as, “*The interaction with people*” and “*The biggest thing to me is that I get to work with all the employees on the feed yard*” highlighted the sense of community and teamwork on the feedlot. One participant described how the small, family-oriented environment contributed to job satisfaction, saying, “*I’d say a good thing about this [feedlot] is just how small and family-oriented it is. It’s just like, I mean, if you need time off or something, they’re really flexible and help you a lot.*”

Other recurring themes that shaped job satisfaction included Animal Health and Veterinary Care, Continuous Improvement, and Physical Environment, along with less frequent but significant themes such as Finance, Resources, and Background and Experiences.

#### Pride in job

The Purpose and Role in Job theme was the most prominent when respondents reflected on what made them proud of their work. While this theme included a breadth of sentiments from respondents—including but not limited to satisfaction in problem solving and executing plans successfully, contributing to a team and/or the larger industry, and feeding people and the world, the commonality between most responses coded with this theme was that they almost always included the word pride or prideful. Some of the most common responses shared revolved around providing a safe and wholesome end-product and feeding communities. Examples include, “*I’d say just being a part of helping supply food to the world and trying to raise quality cattle.*”*;* “*There’s always an element of pride that you have in the product and the beef. You know, that’s probably the biggest one that you’re doing work that is noble because you’re feeding people.*”*;* and “*Well, the proudest thing for me is, um, riding the horse, moving the cattle, and seeing the results at the end when the cattle leave the pens for sale. It’s what makes me feel the proudest because I know I’m doing a job, and not only that, but a meal is going to homes for families.*”

This theme was frequently paired with Stockmanship and Stewardship, reflecting the pride workers took in the quality of animal care they provided (e.g., “*Taking good care of the cattle at all levels. From the time they arrive to the time you ship them. If you feel you’ve cared for them, it’s a source of pride.*”). This sentiment was exemplified over and over again by focus group participants, including one respondent who shared, “*One is knowing when we go home at the end of the day, we’ve done everything we can do to be animal caretakers to the best of our ability.*” In another focus group, one person shared quite simply, stating, “*Well, just knowing that you get to take care of something*.”

Relationships also played a significant role in workers’ sense of pride. Comments related to Relationships often emphasized the sense of community at work, with teamwork and shared responsibility in problem-solving being highlighted as key sources of pride. One participant in a focus group illustrated this by saying, “*I’m proud, really every day, of the job that we do, the crew that we have, the system that we have, the performance that we have, I mean not just by the people, but to get the cattle to perform. And, so, that’s what keeps me going*.” Another person echoed this sentiment, saying, “*My pride would be [that] we’re a team here all together, and what we get accomplished every day is, is fulfilling to me*.” In a more detailed account, one respondent shared how the supportive and positive work environment motivates him to be a productive team member, filling him with pride. He expressed, “*What makes me feel proud to come to work here, the environment where I develop. It is an atmosphere of cordiality, especially with the managers, with the employers, with the bosses, it is an atmosphere of cordiality, full of trust, and positive energy, which makes coming to work not a burden. It is to be able to give them support, to be able to repay them for the support they are giving us, in this case…the support they are giving me, giving me the opportunity to work for them. It is to repay them in a good way. And what makes me feel proud? That…every day they have given me the confidence to come to work, so I have to respond to a daily challenge, constant, but with a positive energy thanks to the feeling and the atmosphere that takes place here*.”

#### Negative attributes and job-related challenges

When discussing the least enjoyable aspects of their jobs, respondents frequently pointed to the challenges posed by the Physical Environment and Resources. Weather-related issues, including extreme heat, storms, snow, and mud, were a common source of frustration for workers. One respondent remarked, “*I would say it’s tough working out in the conditions sometimes*,” while another stated, “*We battle a lot with heat, storms, snow, snow blizzards. Those are never any fun*,” highlighting how unpredictable and extreme weather made it difficult to perform work efficiently. Similarly, many workers expressed concern about how weather conditions impacted animal welfare, with one participant saying, “*Probably the location where we’re at, we get winter and summer. So, it’s cold, muddy, six months out of the year, and then it’s dry and hot. Dusty. The main thing is that we’re all worried about the cattle suffering from both. It’s probably the hardest for me anyways to see the cattle like that*.” Respondents also shared concerns about the toll that challenging weather conditions took on their own well-being. One worker commented, “*On those like 110-degree days, trying to keep cattle alive as well as keeping yourself alive and not passing out. And then in the wintertime, yeah, checking cattle in the freezing cold and snow and spring and kind of depressing weather. It can take a toll on you, but yeah, I’d say kind of the variable weather is kind of one of my least favorite things about it*.”

The resources theme was evident as another significant concern, with workers discussing the challenges posed by insufficient resources, such as labor shortages, inadequate facilities, and equipment breakdowns. Availability and quality of labor were frequently mentioned by respondents, including comments such as, “*I think what we’re seeing…is just with the workforce, the higher turnover, the accountability has kind of changed. So, you know, the calling in, showing up late, like just those kinds of things. It’s different*.” and “*Probably one of my least favorites is the struggles we have with employees. I mean, and not necessarily with the employees we have, but retaining employees, and it’s just hard to find quality employees now, and I think that’s one of the worst things I, that I feel like that I have to deal with*.” Additionally, other respondents expressed challenges with equipment breakdowns, which they described as inevitable but nonetheless challenging (e.g., “*Every job entails problems. I mean it seems like every day there’s something broke down…*”). When speaking about resources, some respondents mentioned that there are new or different management practices they should practice, which often require extra time and effort to implement. An example of this includes, “*What I don’t like is… the lack of preparation or awareness of cattle management, that’s the only thing. We are all aware and we all know how to do it, but there are improvement techniques that we could apply, that is the only thing. But it would be a matter of training talks, nothing more*.”

#### Improving the job

When asked about what could make their job better or easier, respondents overwhelmingly discussed the importance of Resources, focusing on facilities, labor, and technology. Many participants emphasized that better staffing would ease the workload and improve operational efficiency, with one respondent stating, “*Just to be fully staffed every day, I think would be huge.*” and “*It just seems like, culturally, people’s work desires and what they want to do is changing…It makes it tough*.”

Another aspect of Resources was technology, with respondents expressing both enthusiasm and frustration about its potential. Some workers were optimistic about automation improving efficiency, with one sharing, “*The first thing that popped into my head was if we could automate a few things to make everybody’s job just a little bit easier. It would actually affect multiple departments. Get rid of papers to sign and make it more computer. So, it’s like once you’re done with the process, you’re already entering stuff. Instead of having to go print a paper, sign it, hand it in, and then worry about did they put the date right? Did they sign it? Did they forget to sign? It’s just done right there in front of you at the time of the event. From quality assurance and control stuff, that would make, I think, things cleaner.*” However, some respondents thought technology could make their job harder, especially if it is not integrated well across departments and different operating systems. One respondent stated, “*That’s kind of like my frustration point right now [with technology] is that they’re trying to take [it] a step forward, but we mostly just keep stumbling over ourselves. Like we just got the new tablets and all this new software and there’s all this new computer stuff—which I’m sure six months from now we’ll look back and be like* “*oh yeah this was a great switchover*” *– but like right now it’s just super frustrating…*”. Additionally, generational differences arose in conversations surrounding technology, as younger respondents were typically more apt to welcome and use new technologies with greater ease, in comparison to older respondents, who often shared their struggles about learning and adapting to new systems.

Facility maintenance and infrastructure improvements were also commonly mentioned. One respondent highlighted the challenges of working with outdated equipment, saying, “*We’re currently working with the aging, failing system that we’re making work while we build a new facility—state of the art. So, we’re looking forward to that as one of the advantages [the new mill] will bring.*” Another emphasized the impact of broken equipment on daily operations, saying, “*Anytime gates are not working right or the fence is not working, it’s hard to pull cattle out of a pen if you don’t have a gate to get them out.*” One respondent simply stated that “*Less breakdowns*” would make their job easier.

Finally, respondents shared feedback about the need for new educational opportunities and assistance. Specifically, one respondent mentioned that language classes would be beneficial to them, sharing “*I guess if I got Spanish speaking courses. It would make more sense for me to learn Spanish than 30 people to learn English. So that would make my job a lot easier*.”

### Related to training, cattle care, and animal well-being 

This block of questions focused on two areas: learning and training (five questions) and cattle well-being and cattle care challenges (five questions; Table 1). The learning and training questions explored what participants knew about cattle care before starting their jobs, what they have learned since, how they acquired that knowledge, whether they found their training helpful, and whether they desired additional training or resources.

#### Learning about cattle care

Participants were asked about what they had learned regarding cattle care since starting their jobs, and several key themes were identified in their responses, with Animal Health and Veterinary Care, Stockmanship and Stewardship, Continuous Improvement, and Providing Opportunities for Good Welfare and Positive Experiences being the most prevalent. These themes were present to varying degrees, with Animal Health and Veterinary Care being the most commonly mentioned.

Animal Health and Veterinary Care encompassed comments about identifying and/or treating sick animals (e.g., “Diagnosing sicks.”), administering medicines or other vet care, and meeting withdrawal times. Comments may also have included discussion about performance or productivity related to animal health (e.g., performance lost because of disease). Speaking about identifying and treating sick cattle, a commonality throughout many focus groups, one respondent shared, “*I have learned to treat and differentiate a [sick] cow. It depends on what disease it has; they can contract many types of diseases. That is what I have learned.*” Echoing the previous statement, another respondent stated, “*Well, what I have learned here, to implant, before I didn’t know how to implant. Well, the same in the handling of medicines, here I have learned a little more…here they teach you a little more about medicines, and which ones work better for the animals.*”

Furthermore, many participants discussed advancements in animal health technologies and the hands-on learning they experienced. As one respondent explained, “*You know, the guys out on the pasture, they might get a new medicine now and again that their boss tells them about, but in here you get to see the reps, you get to go to the meetings, you get to actually see the bar graphs and see this saves these lives and this one doesn’t, you know, and you just learn a whole lot more here just because, like I said, you’ve got so many more cattle to take care of*.”

The theme of Continuous Improvement was present in nearly half of all responses, reflecting a strong emphasis on ongoing learning and adaptation. This theme often included discussions about using feedback from audits, assessments, and external consultants to improve practices. One respondent shared, “*And then what I know today about nutrition and health, I mean, those are the kinds of things that I didn’t know any of that then. But now I would say that because of all the time spent with consultants and that over the years, I kind of know what the right thing to do is.*” One respondent’s comment underscores how third-party audits and technological advancements have played a crucial role in this process. One respondent shared, “*I think, you know we do a lot more animal well-being and third-party auditing, loading, you know everything that we do. What’s changed the most is record keeping is easier because it’s all computerized now and back in the day we used to have to look through note cards and find the animal. And then you have to figure the days and this and that and now it’s all just on there. I think that’s probably been the biggest, technology. It’s made things a lot simpler.*” Comments coded with this theme also reflected a dynamic and ongoing learning processes; this sentiment is captured well in one respondent’s statement about discovering something new daily, sharing, “*And you think you know everything, but there’s something every day.*”

Alongside Continuous Improvement, the theme of Providing Opportunities for Good Welfare and Positive Experiences was also commonly mentioned. Participants noted that ensuring the well-being of cattle is a priority and a continuous focus. One respondent explained, “*And we try to have that fresh feed in there to receive the cattle and it’s how we handle going into that new environment. You know, that has really changed. I mean not that we never tried…I just didn’t know, but that’s because now we have good nutritionists and vets, and the industry is picking up on things.*” Another participant shared how practices had evolved over time to improve animal welfare: “*Even over the last month we’ve bedded cattle because of the heat. It was something that we haven’t done a lot of, but it’s just something that’s been out there and something that we’ve tried. But doing more to make cattle comfortable when they come in is something that’s evolved over the years. There’s some things that are more high-tech and there’s some things that are just animal husbandry. Not new things, maybe even some things we go back to.*”

Respondents also highlighted their commitment to ensuring that cattle are well cared for, with several emphasizing the priority of animal welfare (Providing Opportunities for Good Welfare and Positive Experiences). One shared, “*I would say that [the cattle] always come first. They’re priority one.*” Another respondent noted the importance of providing adequate water and feed: “*Yeah, shade and water. Obviously, you know taking care of their health is very important. Vaccinations are very important. Making sure pen maintenance is really good. I mean there’s so many things. I mean they all have to work together.*” Clean water, quality feed, and a comfortable environment were repeatedly mentioned by respondents in responses to this question (e.g., “*We fix the tanks so that they have enough water for them to drink and have good nutrition. And that the water is clean*”). Additionally, one respondent summed up the philosophy of animal welfare with, “*It’s all about animal welfare—simple. Thing is, take care of the cattle, they’ll take care of you. But we’ve took that beyond. We handle lighter cattle here, so we want to be a five-star resort. So, we would have a clean room, bed, fresh water, clean water. We have our pens, you know, they are bedded. We have shades where we have compromised cattle.*”

The Relationships theme was identified less frequently but still provided valuable insights, particularly in the context of managing employees. One supervisor explained how their perspective on working with people had evolved, recognizing that everyone has different ways of working and personal challenges. The supervisor stated, “*For me…just working with people and just realizing that each person is different, and they’re all going to work differently. Also, I don’t know what’s going on at home and so a lot of times I think we’ve all had those days where if you’re stressed about something at work or at home, it’s going to affect your work. I think that’s one of the biggest things I’ve learned is that people are just different in general, and everyone’s got different stuff going on.*”

#### Training types and effectiveness

The themes of Continuous Improvement, Stockmanship and Stewardship, Backgrounds and Experiences, and Animal Health and Veterinary Care were the top four themes coded in responses to questions about training. These findings underscore the emphasis given to animal care and husbandry training in the feedlot industry, as many responses coded with these themes spoke to training regarding animal health, general care, behavior and handling, and feeding practices. Regarding the type of training people have received to do their job, many respondents mentioned BQA Training as a foundational component of their knowledge and training programs (e.g., “*Well, BQA training, everybody gets it on the first day of employment.*”).

Additionally, the Continuous Improvement theme often appeared alongside other themes, such as the Stockmanship and Stewardship theme, which involved respondents speaking about training in the context of animal handling and behavior. One respondent shared, “*BQA has taught us, this is where you need to stand to get cattle to go this way and stuff like that. We’ve been doing it our whole lives, but it’s like, oh, that is a little bit easier. It’s safer for them, safer for us. And some of our goals are to do the actions that we do to the cattle, as far as treatment, without affecting their lives. Affecting them very little.*” Echoing the previous statement, but also speaking to the Animal Health and Veterinary theme, another person stated, “*[Our BQA Coordinator]…will come in and watch slips and falls and catches and the process and you know, the misses of what we do, and that is something I have learned—we used to try to catch the cattle and if we missed them, hold onto them as long as we could to get the stuff done. Well, it is better to let them out and re-catch them—you know, that is stuff that you asked on the prior question about what we have learned, that is stuff we’ve learned in the past. Just administering medicines also and where they are effective and not—you know, the modified lives—mixing those and not opening the container and dumping it in. Just being more efficient and effective in what we’re doing. If we are going to be doing it, to do it right.*”

While animal care and husbandry were dominant and recurring concepts in conversations about training-related questions, people management also came up. Respondents, particularly supervisors, mentioned engaging with consultants to improve their skills in managing and communicating with their teams. One respondent shared, “*We had a guy come in, he was one of the founding vets on the program that we use, and he taught us a lot of things and trained us on how to handle people, you know: [*“*Hey, why don’t you give them ownership over this? They are saying it. Give them the tools to go do it. And let them do it because they will take ownership over it, and they will be happier*”*]*.” Across focus groups, there was a strong emphasis on the need to tailor training to accommodate different learning styles for example: “*I’ve noticed that a lot of people here are visual learners. I’m a visual learner. If I don’t know why I’m doing it or if I don’t practice it enough, it’s not going to sink into my brain. So, a lot of times when I train people, it’s like, okay, cool, we ran through that demonstration on the screen, but let’s go put it in motion real quick.*” People also repeatedly mentioned learning from their colleagues, which often happens informally but has a great impact. Respondents shared statements such as, “*I think that there are several factors because, in fact, they give us classes, small groups, veterinarian training and you learn in different ways, but you learn. Sometimes you learn, for example, from other colleagues*.” and “*I rode with him for a while. He showed me, and I’ve been here for two and a half, three years, and he showed me some tricks in two weeks riding with him that I have a problem with my horse. You can learn a lot by talking to your other cowboys on how you’re handling your cattle, if you’re having a rough time, if you’re having a rough time with your horse or anything…If you just kind of talked to them and listened to other people, you can learn a lot by just listening and paying attention to what other people do.*”

The Backgrounds and Experiences theme was frequently identified in responses to training-related questions and was almost always coded with the Continuous Improvement theme (e.g., people spoke most often about how their backgrounds and previous experiences influence the way they learn). For example, one person shared, “*Well, part of it is what I already knew from my work in Mexico, part of it is the experience of others who teach you how to work, it’s all part of it in this job.*” Speaking more to their backgrounds and experiences, another respondent shared, “*I really liked watching cowboy movies. Whenever I went to the movies, I always liked…to look for the movies that were about cowboys. And I always liked that. Yeah, but, well, that’s more or less why I also had an idea, right, of how to manage cattle because it’s not the same, right, to see it, than to do it. Because there is a big difference from, from seeing to being, to getting into it and to what is real about work. To have the knowledge and experience of what it is like to work with the cattle.*”

#### Animal well-being

Cattle comfort was a recurring concept in answers to animal welfare-related questions, which was coded under the Providing Opportunities for Good Welfare and Positive Experiences theme. Aspects of comfort included a comfortable, clean, and dry pen with ample space to move around, fresh water, and quality feed. Speaking to Providing Opportunities for Good Welfare and Positive Experiences, but also to the Stockmanship and Stewardship theme, one respondent shared, “*I’d just kind of say a little bit like the cardinal rule, but instead of treat people how you want to be treated, treat cattle or animals how you’d want to be treated. Clean pens, fresh feed, fresh water, not abusing them as far as handling wise. Kind of like what I was saying, calmly and respectfully I guess would be what I’d say.*” Echoing the above comment, another person stated, “*I guess all around, mainly their environment, because they’re in those pens all day long. And so, they’ve got proper water, proper feed, shade.*” Some respondents spoke more broadly about cattle comfort, e.g., “*Making them as most comfortable as possible up until the day they go out.*”

While many answers coded with the Stockmanship and Stewardship theme spoke specifically about animal handling and behavior (as shown above), many responses coded with this theme also spoke generally to good animal care and husbandry, and about treating the cattle with respect throughout all phases on their journey in the supply chain. Examples of this include, “*Well, I’ve worked around cattle all my life and there are some people that just think the meaner they get, you know, they can make a cow do what they want. They’ll, you know, over abuse them when they don’t have to. You know, respect them. We ain’t gotta be mean to them to get them to do what we want them to do. Just overall stewardship, good stewardship of the cow.*” and “*It means taking care of cattle where they’re intended to be taken care of.*” Lastly, one person shared, “*I would say, what does cattle mean to me? I would say, like if I was a cow, then I would treat it by taking care of them the way I would want to be treated. Because if you’re taking care of them, they’ll take care of you.*” The respondent continued, “*I think it’s very critical though in the feed yard, especially because that’s the last days before they go to the meat or the packer house. So, it’s very critical.*”

Speaking about animal welfare in context of the physical environment was also a commonality between focus groups—the Physical Environment theme was mentioned in one-quarter of all responses. Discussions around caring for cattle in different elements and conditions, such as the heat, rain, cold, and wind, were frequently mentioned. For example, “*Comfort. They’re a big animal. They shouldn’t be miserable, you know, they should not be miserable. I know we just got two inches of rain. So, it’s going to be muddy, but you know they shouldn’t be without feed and water. They shouldn’t be standing in holes. We put them in that situation to be in here…so, make them as comfortable as they can be.*” Another respondent shared, “*Like I said, cattle get put into environments, geographically, regionally, you know the logistics that goes on with it…we know we don’t live in a perfect world—and we know that. If I could just change one thing, make the weather just perfect. 70 degrees during the day and 45 degrees at night and cattle love that environment, but we don’t live in that. So, if we can find, you know animal welfare is one of the first and foremost things that we need to look at.*”

Cattle well-being was often discussed as a shared responsibility, which requires accountability from everyone on the feedlot; one person said, “*So, if you do your part, and another person doesn’t do it, then you won’t see the relaxation that an animal should have*,” continuing, “*It’s because, when cattle arrive at a corral, it depends on many departments. It’s not just the maintenance guys, or the yard guys, it’s not just the equipment guys, it’s not just the feed guys, it’s all of us. There are those who are dedicated to, to medicine, to check them, so they aren’t sick. They also have to have their system of entering the corral, how they are going to enter, how they are going to handle the cattle, like now that the temperatures are high. They have to check all of that. If they start chasing them, of course they’re going to stress them out, they’re going to agitate them.*” Expanding more on the shared responsibility that comes with taking care of cattle, one person shared, “*Know that we then we are feeding the cattle, it’s for one reason. That we all work for one purpose, the more important thing here is to keep those animals in a good place, keep them comfortable. We know what the purpose for it is. That’s something that we have to do.*”

### Related to work environment and community

Participants in this section responded to six questions about their work environment and community (Table 1). These focus group questions focused on what employees valued about their work environment, how they characterized their workplace community, and their preferred methods of communication (under both normal and challenging conditions). The following narrative summarizes key themes that were present in responses to these questions.

Respondents frequently discussed the importance of Relationships when reflecting on their work environment. Positive interpersonal dynamics within teams and between colleagues and supervisors was a key factor in job satisfaction. Words like “family,” “team,” and “camaraderie” were commonly used to describe the work environment. One participant emphasized the importance of teamwork by sharing, “*The way we all, like especially cowboys and processors, especially on our side, how we kind of work together*,” continuing, “*You know, if we’ve got cattle running all over the damn place and [name] is up top and his guys are up top, they’re going to stop and help us. And if they’re having trouble getting fat cattle pushed in to go ship, we’re going to come help. You know, we help each other.*” Speaking about the guys on his cowboy crew, one respondent shared, “*These are good guys. You know, and that’s why we all get al.ng pretty good. And that’s what I always say, you can’t really, if you’re having a bad day or whatever, you can’t say, well, it’s this guy’s fault or it’s that guy’s fault, because it’s not really anybody’s fault. You know, I know a lot of people like to blame…I mean, a lot of times it has to do with the fact that it’s dark, the fact that the wind is blowing, and the fact that it’s muddy and slick—or the fact that this gate doesn’t work.*” One respondent shared more about responsibility, stating, “*We take the work with a certain sense of humor. But responsibility comes first.*”

Respondents also shared a sense of belonging within their workplace. One participant noted, “*You’re not just in that case, I’m talking like if any of us arise with personal issues and stuff like that, I mean not just us being [from another country]. Everybody’s very respectful and very caring it really makes you feel, okay*, “*I belong*”.” Another person shared, “*There’s still a family component to the business, right? That’s helpful. I mean, it gives people a sense of belonging, and also kind of being family oriented and not trying to feed a lot of shareholders their investment in the company for the long term, for the next generation, tools needed, things like that.*”

Laughter, lightheartedness, and fun in the workplace were also mentioned frequently as key aspects of the work environment. One participant shared, “*I’ve always said that I like to have fun. I like to joke around. I have to make my job halfway fun or it ain’t worth doing it.*” Another person echoed this sentiment, saying, “*Yeah, that we all, for the most part, actually get al.ng, joke around. Sometimes it doesn’t even feel like we’re working.*” However, respondents acknowledged that disagreements may arise but are usually overcome with humor (e.g., “*Well, I think that like I was telling you a moment ago, that, as in any family, sometimes one is not in the mood and, well, we get here to, even get angry with each other. Well, after a while it passes, and you go around laughing and laughing.*”; “*I think it’s pretty lighthearted. A lot of people poke fun at each other. Playful from top to bottom. I see that with my guys too. Some of the aspects of the job are tough. Most people try to make light of it and not get super upset or frustrated. For the most part.*”).

The Purpose and Role in Job theme intertwined with Relationships when respondents spoke about their understanding of their role within the larger feedlot and beef industry. Many workers reflected on how their work contributed to the greater mission of the feedlot or industry, providing motivation and a sense of significance in their roles. One participant stated, “*You don’t ever feel like you’re just another cog, I guess. I mean, even if you do a little thing, they make it, you know, that you’ve made a difference.*” Another shared, “*But in the end we are family and here we are and we all unite because I believe the same, the work for, for what we do.*” Respondents also expressed satisfaction in working together to accomplish tasks as a team, with one person noting, “*To me what I like the most is when they put us to do group projects, that we all put ourselves between all of us here and there, and cutting, and doing, and you feel the harmony. You can feel the group, well, that we are giving it everything.*”

Less commonly mentioned but still important, Communication Successes appeared in the context of the work environment. Communication Successes were often related to clear instructions, trust from supervisors, and efficient information sharing among team members. Respondents appreciated the effectiveness of their workplace communication. While most people spoke about helping each other out and working together to get jobs done, some spoke about a lack of cohesion across the feedlot, particularly across different departments on the feedyard (e.g., “*Departmentalized. That would be the best way to put it. As far as every crew just thinks about their job. Instead of the whole.*”*).*

The Physical Environment theme was also discussed, with respondents noting how being outdoors and working in the open air contributed to a positive work atmosphere. One participant shared, “*Walking outside, walking in the fresh air and not being cooped up in a room all day. Many jobs. No more lazing around in a room for many hours. And when an afternoon comes, the person grabs air, grabs dust, grabs whatever he grabs, but feels more at ease. Freer. Freer.*” Additionally, some workers appreciated the solitary nature of certain tasks, with one person saying in reference to driving a feed truck, “*I’m in my own sanctuary.*”

## Discussion

Feedlot workers play a critical role in ensuring the health, performance, and well-being of beef cattle raised in feedlots, yet their perceptions have gone largely underrepresented in the scientific literature. To the author’s knowledge, only one study to date has directly evaluated the perceptions of feedlot workers regarding job satisfaction and topics related to animal care (Ridge et al., 2019). A small number of related studies have examined similar populations, though they have primarily focused on occupational stressors and workplace safety (Ramos et al., 2018, 2021a, 2021b; Carlo et al., 2023), with limited attention to the intersection of job satisfaction, cattle care, and workplace community. In the current study, focus groups were used to explore the experiences and perspectives of feedlot workers and to provide insight into how these individuals understand their roles, responsibilities, and relationships with the cattle in their care and the people they work with. Across all focus groups, respondents expressed a strong sense of pride and responsibility in their work, emphasizing both their commitment to cattle health and well-being and to producing safe, high-quality beef.

The participants in this study were representative of the United States agricultural workforce. Although agricultural employee statistics are not reported by livestock sector, the Bureau of Labor Statistics (BLS) 2024 survey of agricultural and related industries reported that the majority of wage and salary workers are men between the ages of 25 and 45, statistics that are in line with the current study (BLS, 2024a). Just over half of the current study population identified as Hispanic. The BLS reported that people employed in animal production and aquaculture were 20.5% Hispanic or Latino (BLS, 2024b); this is less than reported in the current study but the BLS information includes all types of employees (i.e., owners, self-employed, wage workers, etc.). Type of work (seasonal vs permanent) and/or visa status was not asked in this study but could be considered in future work as there is limited research in this area and previous has suggested that worker status can impact job satisfaction of employees (Hobbs et al., 2020; Ramos and Reynaga, 2023). Feedyards in 11 states were included in this study, representing several of the states with the highest numbers of cattle on feed (e.g., Texas, Nebraska, Kansas; USDA NASS, 2025). Future efforts should aim to include regions that were less well represented—particularly the Southeast. Operations in this region were more difficult to recruit for the current study but remain an important segment of the industry and should be prioritized in future research.

### Related to job satisfaction

Findings from this study highlight the central role of pride, responsibility, and interpersonal relationships in feedyard employees’ perceptions of job satisfaction. Participants repeatedly referenced their enjoyment of working with cattle, the personal fulfillment derived from contributing to the beef supply chain (particularly through the act of feeding people), and the importance of teamwork and workplace camaraderie. Additionally, stockmanship and stewardship were not only central to the daily tasks employees performed, but also closely tied to their sense of pride, professional identity, and responsibility. This finding aligns with previous research emphasizing the relationship between human–animal interactions and meaningful agricultural work (Hemsworth and Coleman, 2011; Edwards-Callaway and Calvo-Lorenzo, 2020). Additionally, similar themes were observed in focus groups with cattle transporters, where participants expressed a strong sense of pride, responsibility, and care in their work (Sullivan et al., 2025). Across discussions, transporters consistently emphasized that working with cattle and ensuring their well-being was central to how they defined success in their jobs. Findings from both the current study and Sullivan et al. (2025) suggest that pride in work, responsibility for animal welfare, and meaningful human–animal interactions are key motivators for cattle caretakers and that feedlot employees derive meaning and job satisfaction from fulfilling their roles as good stewards of the cattle in their care.

Quantitative results from this study support the thematic analysis related to job satisfaction, with nearly all respondents indicating that they take pride in their work and are satisfied with their current responsibilities. These findings are consistent with previous research showing that pride and a sense of task significance are important motivators in emotionally demanding professions (Lewig and Dollard, 2003; Jin and Guy, 2009). Although livestock work is not traditionally considered emotional labor, caring for cattle requires managing both physical demands and personal emotions, especially when working in fast-paced, high-pressure environments. There are parallels in other animal care-related fields, primarily the veterinary profession; animal care work can be highly stressful and emotional (Scotney et al., 2019; Thompson-Hughes, 2019; Wallace, 2019) but studies have also shown that this type of work also brings pride (personal and professional), job satisfaction, and gratification (Clise et al., 2021; Deacon and Brough, 2021; Elte et al., 2023; Whitaker et al., 2025). In a study on veterinary nurses’ caregiving experiences in companion animal medicine, participants shared that getting through the sometimes very difficult situations gives them a sense of pride in their own and the profession’s resilience and positively influences job satisfaction (Deacon and Brough, 2021). An international study on equine veterinarians also demonstrated that pride and purpose influenced work engagement and satisfaction (Elte et al., 2023). Providing meaningful care for animals can enhance a worker’s sense of purpose and fulfillment (Polachek and Wallace, 2018; Wallace, 2019; Sullivan et al., 2025); supporting this, a survey of Texas feedyard workers found that nearly half of respondents entered the profession because they enjoyed working with animals, highlighting the intrinsic motivation that animal care brings to cattle industry employees (Ridge et al., 2019). Workplace culture, particularly the strength of interpersonal relationships, also emerged as a major contributor to job satisfaction. Respondents in the current study described a sense of camaraderie, humor, and mutual support that made challenging work environments more manageable. These findings are consistent with previous literature identifying social support and positive workplace dynamics as important protective factors against occupational stress (Ramos et al., 2021b; Garmendia et al., 2023). Both company and team culture and environment have been shown to influence equine (Elte et al., 2023) and companion animal veterinary health-care professional job satisfaction (Moore et al., 2014). Transporters in the Sullivan et al. (2025) study shared similar sentiments regarding team culture, which they reported as being an aspect of the work environment they enjoyed the most (e.g., “*We have each other’s backs. It is like having a really big family*” and “*I like the quality of my coworkers—the characters in most of my coworkers, compared to other jobs I’ve had. And of course, I love working with animals, but I think anybody that loves working with animals is gonna be a little better character. And I noticed that I like it*”, pg. 5). In the current study, participants frequently cited the importance of working alongside colleagues who share similar values and work ethics, fostering what several focus group participants described as a “family-like” or “team-oriented” environment. Previous studies of farmers and livestock caretakers similarly emphasize that good team spirit and social cohesion are highly valued intrinsic motivators (Kolstrup, 2012; Muri et al., 2020). This emphasis on workplace relationships is further supported by Ridge et al. (2019), where workers highlighted teamwork as essential for maintaining morale, even when facing the demands of long work hours and physical challenges. Brando et al. (2023) in a survey on job satisfaction and workplace stressors in zoo and aquarium professionals reported similar sentiments; participants identified collaboration in animal care and trust between colleagues even when their job was not always easy and sometimes overwhelming.

In addition to strong social bonds, respondents frequently mentioned the autonomy, skill variety, and purpose inherent to their roles as sources of pride and satisfaction. These attributes align closely with Hackman and Oldham’s (1976) job characteristics model, which posits that autonomy, task significance, skill variety, task identity, and feedback are key drivers of job satisfaction. Participants in this study emphasized their ability to make meaningful contributions daily, engage with a variety of tasks related to animal care, and experience direct feedback from both animal outcomes and team dynamics. Additionally, the ability to work outdoors, interact with animals, and take pride in daily accomplishments reinforces the intrinsic appeal of feedyard work for many employees, which aligns with findings among other agricultural populations (Kolstrup, 2012; Muri et al., 2020; Sullivan et al., 2025).

However, despite these positive aspects, several challenges were identified that could diminish job satisfaction over time. Persistent obstacles related to the physical environment, including extreme weather conditions (e.g., mud, heat, wind, and cold), were commonly mentioned, as were frustrations stemming from labor shortages, equipment failures, and aging infrastructure. These challenges mirror findings from previous work demonstrating that operational and environmental stressors significantly affect employee satisfaction and animal care practices (Sullivan et al., 2025). While participants expressed resilience and adaptability in responding to these challenges, there was clear consensus that consistent labor availability, preventative equipment maintenance, and structural improvements would ease workload stress and enhance both employee well-being and cattle welfare outcomes, an area of need that should be addressed. Recognizing that team meetings alone won’t resolve these multifaceted issues, using them to discuss potential solutions is beneficial for brainstorming, understanding the perspectives of those directly affected, and, importantly, showing employees their opinions are valued.

Notably, agreement with statements related to job pride and feeling valued differed by preferred language, with Spanish-speaking workers less likely to report strong agreement. This finding highlights the importance of culturally inclusive workplace practices. Previous research has shown that language barriers can limit access to training, recognition, and advancement opportunities, ultimately influencing both satisfaction and retention (Justen et al., 2009; Valdivia and Flores, 2012; Román-Muñiz, 2023). Ridge et al. (2019) similarly identified role-specific knowledge gaps and training challenges among diverse feedyard workers, suggesting that differences in background, language, and access to information can significantly impact employee confidence and satisfaction. Integrating bilingual training programs, ensuring clear communication across all workforce tiers, and fostering inclusive management practices are critical steps for supporting all employees equally and sustaining high standards of cattle care (Calvo-Lorenzo, 2018). Industry groups have been proactively adapting both educational and training materials into Spanish to increase accessibility to a larger group of stakeholders and users (The Meat Institute, 2021; The National Dairy FARM Program, 2021; personal communication, NCBA). Taken together, these findings suggest that feedyard employees derive significant meaning and satisfaction from their work, stemming primarily from positive human–animal interactions, pride in task accomplishment, and strong workplace culture and relationships.

### Related to training, cattle care, and animal well-being 

Themes related to training, learning, and continuous improvement were prominent in participants’ responses, particularly in the context of cattle care and well-being. While formal training programs, such as BQA, were consistently mentioned as important, informal and experiential learning through mentorship, peer to peer learning, and daily practice played a significant role in how employees developed and applied their skills. These findings emphasize the need to design training strategies that not only reflect how learning occurs on feedlots, but also ensure training is accessible to a diverse workforce. Consistent with previous research on adult learning in agriculture, participants emphasized the importance of hands-on demonstrations, visual instruction, and opportunities to practice skills in real-world settings (Edwards-Callaway et al., 2024; Sullivan et al., 2025). Several respondents also noted that bilingual instruction is critical for supporting Spanish-speaking employees, reinforcing the importance of culturally inclusive workforce development.

Ensuring animal welfare throughout the livestock production chain is a shared responsibility across all sectors, from farm to table, and an increasingly important consumer expectation (Busch and Spiller, 2018; Yang and Renwick, 2019). Animal welfare is inherently complex and multifaceted, often resulting in diverse perspectives among stakeholder groups regarding what constitutes good welfare (Vanhonacker et al., 2008; Degeling and Johnson, 2015). Findings from this study indicate that feedyard employees who directly manage animal care demonstrate a clear understanding of animal welfare within the context of their daily responsibilities and the vast majority of participants agreed that “Animal well-being is a critical component of [their] job.” Notably, the concept of providing opportunities for animals to experience positive welfare states emerged frequently. Although feedyard employees did not reference specific welfare frameworks (e.g., Five Domains Model), their comments mirrored a shift seen in welfare assessment, that is, providing animals with positive experiences and opportunities in their environment (Five Domains Model, Green and Mellor, 2011; Five Opportunities to Thrive, Greggor et al., 2018) and highlights an encouraging shift in how feedyard employees conceptualize cattle welfare. This attention to providing positive experiences for the cattle they care for aligns with the prior discussion around being prideful in their role in providing animals with a good life.

Predictably, animal health was consistently recognized as integral to welfare and identified as a significant focus area for training and knowledge acquisition. Maintaining animal health is foundational to daily feedyard management, explaining its prominence in participant discussions. Their frequent mention and apparent adherence to the BQA program likely also correlates with their emphasis on animal health, given the program’s strong focus on health prevention and management. Additionally, a strong commitment to continuous improvement was evident across focus groups, with many individuals expressing interest in expanded training opportunities, particularly hands-on and experiential learning, to further develop their skills. Implementing collaborative or team-based learning opportunities have been shown to enhance engagement, communication, confidence and teamwork across both veterinary and agricultural applications (Mills, 2003; Dale et al., 2005; Hazel et al., 2013; Edwards-Callaway et al., 2024) and thus would be valuable to incorporate into feedyard training programs. Providing and encouraging employees to pursue activities that enhance personal and professional growth impacts job satisfaction (Elte et al., 2023). This clear desire for more learning and educational material is an opportunity for the BQA program to deliver more continuing educational programs that have a broad reach across regions of the United States. Although delivering content in-person potentially requires more time and resources, it was evident from the focus groups by the examples given by participants that the visits from professionals (i.e., veterinarians, BQA coordinators, and consultants) were influential to their cattle care knowledge acquisition.

One of the most common animal welfare challenges mentioned in the focus groups was the impact of the physical environment, that is, the snow, rain, mud, and heat. As noted, the physical environment was also identified as a job challenge due to its impact on the workers themselves as well. Climatic changes can directly (e.g., metabolic alterations, immune suppression, and death) and indirectly (e.g., vector-borne diseases, mycotoxins in feedstuffs) impact livestock welfare (Lacetera, 2019). Although heat stress is not the only type of environmental challenge, it is one that has received considerable focus in the cattle feeding industry in part due to the increase in extreme heat events in the summer months that many feedyards have been experiencing (Farm Progress, 2011; Carr, 2017; Guilfoil, 2022). A survey of feedyard veterinarians, nutritionists, and operators reported that participants identified both the physical and emotional impact of working in heat stress conditions when the welfare of the cattle is severely impacted (Dean et al., 2023). Climate variability is a challenge for cattle feeding systems globally and exploring methods to mitigate heat stress is a priority for feedyards across multiple cattle-feeding regions in the United States. Caretakers play a critical role in executing heat stress management plans and recognize the importance preventative measures are in maintaining cattle welfare.

### Related to work community and the environment

As already mentioned, the work environment of the feedyard, particularly the relationships with teammates, played a large role in participants’ job satisfaction. Consistent with findings from Sullivan et al. (2025), respondents frequently described their teams using terms such as “family,” “team,” and “support,” indicating strong interpersonal cohesion within departments. In physically demanding and often high-stress environments like feedlots, this social support likely plays a protective role against occupational stress and burnout. However, some participants noted a lack of cohesion across departments, describing a sense of “departmentalization” that sometimes hindered operational efficiency and overall satisfaction. The existence of organizational silos is not unique to feedyards and silos can hinder operational performance, goal achievement, and internal communication (Dell, 2005; Rosen, 2010; Bento et al., 2020). In his discussion of water and wastewater utilities, Dell (2005) addresses the importance of focusing efforts on the performance of end-to-end business processes (i.e., the complete process of starting an objective from start to finish) instead of functional objectives (i.e., specific tasks or functions). Dell (2005) elaborates to identify that the challenges faced by utility companies are complex, solutions lie across departments, everyone must work together with the same end goal, and everyone must be respected as a part of the process. Although this work was conducted in a different industry, it draws several clear parallels to feedyard operations. Like other animal production systems, ensuring cattle welfare is a complex process that requires coordination across multiple departments—including feeding, shipping, maintenance, and animal health. Participants in this study echoed this idea, emphasizing that animal care is a shared responsibility across the yard. However, several noted that this message can sometimes be difficult to communicate effectively within teams. Feedyard management should evaluate ways that they can intentionally try to break down inter-departmental barriers to achieve higher levels of operational efficiency, in addition to improved caretaker and cattle well-being. While within-team relationships were often strong in the current study, findings suggest that there is an opportunity to strengthen communication and collaboration across functional areas of the operation and between employees and supervisors.

Related, participants shared that they valued the team environment of their workplace. Operations often encourage teamwork but perhaps there are more formal ways to support and promote working in teams focused on cattle care outcomes. Condly et al. (2008), in a meta-analysis of the use of incentives to motivate performance, reported that team-based incentives had a greater positive effect on performance compared to individually directed incentives. Another meta-analysis (Garbers and Konradt, 2014) also demonstrated that team-based incentives have a positive impact on performance. Perhaps there is an opportunity for operations to capitalize on this positive attitude toward working on teams to target specific outcomes related to animal care through team collaboration. It should be noted that research had identified varying impacts (e.g., sometimes positive and sometimes no effect) of other factors such as task complexity, gender heterogeneity of the group, and group size are associated with the effect of team-based incentives on performance (Bowers et al., 2000; Garbers and Konradt, 2014) and thus these factors, in addition to others that may be relevant to this population (i.e., years of experience and preferred language) should be considered when developing incentive programs.

When participants described their work environment in negative terms, it was often linked to ineffective communication or a lack of clarity around roles and responsibilities. While this theme was less prominent in the focus groups overall, about one-quarter of survey respondents indicated that they had difficulty communicating with coworkers or supervisors. Agreement was not associated with age in this study. Although there is evidence suggesting generational differences (generational differences being a proxy for years of experience in this study’s context) in workplace behavior and values (Schullery, 2013), there are also some conflicting opinions about the theoretical foundation of this work and contradictory results (Lyons and Kuron, 2014; Jones et al., 2018). However, a digital divide does exist between older adults and younger generations, and this gap is impacted by many different factors but access, attitudes, self-efficacy are commonly cited (Czaja and Lee, 2007; Charness and Boot, 2009; Tsai et al., 2015). As feedyards transition to more digital communication (i.e., texting and use of messaging applications) in addition to the increased implementation of other technologies for animal monitoring purposes, which was identified in focus groups as a common trend, differences in preferences across individuals within a yard may become more apparent. It would be valuable for feedyards to ensure that all employees feel comfortable with the main methods of communication used on the yard and provide the appropriate support and training to ensure that everyone feels self-efficacious when using the different information and communication technologies.

Language preference was also associated with identified challenges in communication. Although many Hispanic individuals in the United States are proficient in English and/or bilingual (Krogstad and Gonzalez-Barrera, 2015), some do not speak English at all, and others may prefer to communicate in Spanish. There has been some research exploring the importance of language in delivering training materials; the authors recognize that the question in the survey more broadly asked about communication. Ramos et al. (2018) showed that the majority of Latino immigrant feedyard employees preferred to be trained in Spanish. Providing training materials in Spanish is an important step toward improving accessibility for Hispanic workers, but simple translation alone is not enough (Rovai et al., 2016; Sánchez et al., 2019). To be effective, training programs must also be culturally responsive—reflecting the traditions, beliefs, and values of the individuals being trained (Rovai et al., 2016; Román-Muñiz, 2023). It should also be recognized that there is a lot of social, cultural, and economic heterogeneity across Spanish speakers (Ardila, 2020) that should also be considered. Feedyards should take this into consideration when developing training programs but also when evaluating their internal communication strategies. One related point is that while the majority of participants agreed that they were valued members of the team, there were some participants that did not agree with that statement. There was a relationship between preferred language and feeling valued. Although the majority of the sentiments shared in the focus groups reflected a strong sense of belonging, there was a population of individuals that maybe did not share the same positive sentiments. Although not extensively studied in agriculture, research in other industries has demonstrated that a sense of belonging impacts job satisfaction, engagement, and retention (Winter-Collins and McDaniel, 2000; Bilginoğlu and Yozgat, 2022). A sense of belonging and feeling valued is critical to workforce retention (Silver et al., 2024), an important component of a sustainable feedyard system. Promoting strong community culture on feedlots represents an important strategy for supporting both employee well-being and operational success.

The final question of each focus group provided participants the opportunity to share any additional thoughts or reflections. Many workers used this time to express appreciation for being asked about their experiences and perspectives; anecdotally, it was evident during the data collection process, although not asked directly, that the participants in this study appreciated someone asking their opinion about things that mattered to them. Their feedback reinforces the important role that feedyard employees play not only in daily cattle care, but also in identifying practical ways to strengthen workplace practices and cattle well-being. The insights shared through this study contribute to a growing understanding of how job satisfaction, training, workplace culture, and animal care are interconnected within feedyard environments.

## Conclusion

This study is one of the first to examine feedlot workers’ perspectives on job satisfaction, training, cattle care responsibilities, and workplace community. Participants expressed a strong sense of pride in their work, often highlighting the value of contributing to producing food, working with cattle, and being part of a supportive team. These intrinsic motivators were central to how workers described their daily experiences at work and could serve as important focal points for recruitment and retention efforts in the beef industry. While overall satisfaction was high, participants also described consistent challenges, including extreme weather, limited staffing, equipment issues, and communication breakdowns. These stressors impact both people and cattle, underscoring the need for structural support. Many of these challenges are not easily resolved; however, acknowledging them, and committing to improvements in training, resources, and communication, could enhance the daily experiences of feedyard workers and support better cattle outcomes.

Future efforts should build on these findings by evaluating how training approaches, including hands-on, bilingual, and peer-supported models, affect both employee development and animal care practices. There is also an opportunity to develop validated tools to measure job satisfaction, communication effectiveness, and training needs specific to feedyards. Programs like BQA could be strengthened through regular assessment of their impact on employee knowledge, attitudes, and practices. By centering the perspectives of feedlot caretakers, this study provides a practical foundation for aligning workforce development with animal welfare goals. Integrating caretaker well-being into broader strategies for sustainable beef production is not only possible, but it is essential.

## Abbreviations:

BQA: Beef Quality Assurance

## References

[skaf400-B1] Ardila A. 2020. Who are the Spanish speakers? An examination of their linguistic, cultural, and societal commonalities and differences. Hispanic J. Behav. Sci. 42(1):41–61. doi:10.1177/0739986319899735

[skaf400-B2] Bento F. , Tagliabue M., Lorenzo F. 2020. Organizational silos: a scoping review informed by a behavioral perspective on systems and networks. Societies. 10(3):56. doi:10.3390/soc10030056

[skaf400-B3] Bilginoğlu E. , Yozgat U. 2022. Retaining employees through organizational social climate, sense of belonging and workplace friendship: a research in the financial sector. IBR. 52(1):67–85. doi:10.26650/ibr.2023.52.806695

[skaf400-B4] Bowers C. A. , Pharmer J. A., Salas E. 2000. When member homogeneity is needed in work teams: a meta-analysis. Small Group Res. 31(3):305–327. doi:10.1177/104649640003100303

[skaf400-B5] Brando S. , Rachinas-Lopes P., Goulart V. D. L. R., Hart L. A. 2023. Understanding job satisfaction and occupational stressors of distinctive roles in zoos and aquariums. Animals. 13(12):2018. doi:10.3390/ani13122018 37370528 PMC10295341

[skaf400-B6] Braun V. , Clarke V. 2006. Using thematic analysis in psychology. Qual. Res. Psychol. 3(2):77–101. doi:10.1191/1478088706qp063oa

[skaf400-B7] Bureau of Labor Statistics. 2024a. Labor force statistics from the current population survey. Household data. Annual averages. 15. Employed persons in agriculture and nonagricultural industries by age, sex, and class of worker. Available from: https://www.bls.gov/cps/cpsaat15.htm (Accessed May 27, 2025).

[skaf400-B8] Bureau of Labor Statistics. 2024b. Labor force statistics from the current population survey. Household data. Annual averages. 18. Employed persons by detailed industry, sex, race, and Hispanic or Latino ethnicity. Available from: https://www.gov/cps/cpsaat18.htm (Accessed May 27, 2025).

[skaf400-B9] Busch G , Spiller A. 2018. Consumer acceptance of livestock farming around the globe. Anim. Front. 8(1):1–3. doi:10.1093/af/vfx005 32002207 PMC6951871

[skaf400-B10] Calvo-Lorenzo M. 2018. Practical approach and outlook regarding animal welfare concerns related to beef feedlot production. In: American Association of Bovine Practitioners Conference Proceedings, p. 140–148. doi:10.21423/aabp-mpc-2018-2127267

[skaf400-B11] Carlo G. , McGinley M., Maiya S., Ramos A. K. 2023. Associations of work-related injuries and stress to family and youth wellbeing among U.S. Latino/a immigrant cattle feedyard workers. Int. J. Environ. Res. Public Health. 20(4):3361. doi:10.3390/ijerph20043361 36834054 PMC9962635

[skaf400-B12] Carr A. 2017. California heat wave kills thousands of cattle and overwhelms dairy industry. Available from: https://weather.com/news/news/california-heatwave-mass-livestock-cattle-deaths. (Accessed May 29, 2025).

[skaf400-B13] Charness N , Boot W. R. 2009. Aging and information technology use: Potential and barriers. Curr. Dir. Psychol. Sci. 18(5):253–258. doi:10.1111/j.1467-8721.2009.01647.x

[skaf400-B14] Clise M. H. , Kirby N., McArthur M. L. 2021. Is veterinary work more than satisfying? A critical review of the literature. Vet. Rec. 188(10):e77. doi:10.1002/vetr.77 34018567

[skaf400-B15] Condly S. J. , Clark R. E., Stolovitch H. D. 2008. The effects of incentives on workplace performance: a meta-analytic review of research studies 1. Perform. Improv. Q. 16(3):46–63. doi:10.1111/j.1937-8327.2003.tb00287.x

[skaf400-B16] Czaja S. J. , Lee C. C. 2007. The impact of aging on access to technology. Univ. Access Inf. Soc. 5(4):341–349. doi:10.1007/s10209-006-0060-x

[skaf400-B17] Daigle C. L. , Ridge E. E. 2018. Investing in stockpeople is an investment in animal welfare and agricultural sustainability. Anim. Front. 8(3):53–59. doi:10.1093/af/vfy015 32002223 PMC6951904

[skaf400-B18] Dale V. H. M. , Nasir L., Sullivan M. 2005. Evaluation of student attitudes to cooperative learning in undergraduate veterinary ­medicine. J. Vet. Med. Educ. 32(4):511–516. doi:10.3138/jvme.32.4.511 16421837

[skaf400-B19] Deacon R. E. , Brough P. 2021. Companion animal death and client bereavement: a qualitative investigation of veterinary nurses’ caregiving experiences. Death Stud. 45(10):805–816. doi:10.1080/07481187.2019.1696424 31778100

[skaf400-B20] Dean L. , Tarpoff A. J., Nickles K., Place S., Edwards-Callaway L. 2023. Heat stress mitigation strategies in feedyards: Use, perceptions, and experiences of industry stakeholders. Animals. 13(19):3029. doi:10.3390/ani13193029 37835635 PMC10572074

[skaf400-B21] Degeling C , Johnson J. 2015. Citizens, consumers and animals: What role do experts assign to public values in establishing animal welfare standards? J. Agric. Environ. Ethics. 28(5):961–976. doi:10.1007/s10806-015-9584-3

[skaf400-B22] Dell R. K. 2005. Breaking organizational silos: Removing barriers to exceptional performance. J. Awwa. 97(6):34–36. doi:10.1002/j.1551-8833.2005.tb10902.x

[skaf400-B23] Edwards-Callaway L. , Davis M., Dean L., McBride B. 2024. Stakeholder perceptions of animal welfare as a component of sustainable beef programs in the United States—a pilot study. Animals. 14(9):1348. doi:10.3390/ani14091348 38731352 PMC11083033

[skaf400-B24] Edwards-Callaway L. N. , Calvo-Lorenzo M. S. 2020. Animal welfare in the US slaughter industry—a focus on fed cattle. J. Anim. Sci. 98(4): skaa040. doi:10.1093/jas/skaa040 32026929 PMC7134563

[skaf400-B502] Edwards-Callaway L. N. , Sullivan P. A. 2024. The interface of caretaker and animal well-being as a critical component of sustainability. Meat Muscle Biol. 8(1):1–8. doi:10.22175/mmb.18196

[skaf400-B25] Edwards-Callaway L. , Mijares S., Okoren C., Rogers C., Sullivan P., Davis M., Cramer C., Román-Muñiz N. 2024. Developing a model to promote caretaker confidence and communication in treatment decisions for dairy cattle through case studies. J. Dairy Sci. 107(4):2321–2331. doi:10.3168/jds.2023-23698 37944803

[skaf400-B26] Elte Y. , Acton K., Martin J., Nielen M., Van Weeren R., Wolframm I. 2023. Engage and enjoy–investigating predictors of employee engagement and work satisfaction in equine veterinary professionals. Front. Vet. Sci. 10:1036388. doi:10.3389/fvets.2023.1036388 36876013 PMC9975571

[skaf400-B27] Farm Progress. 2011. Heat wave has killed up to 4,000 Iowa cattle. Available from: https://www.farmprogress.com/corn/heat-wave-has-killed-up-to-4-000-iowa-cattle. (Accessed May 29, 2025).

[skaf400-B28] Garbers Y , Konradt U. 2014. The effect of financial incentives on performance: a quantitative review of individual and team‐based financial incentives. J. Occup. Organ. Psychol. 87(1):102–137. doi:10.1111/joop.12039

[skaf400-B29] Garmendia P. , Fernández-Salinero S., Holgueras González A. I., Topa G. 2023. Social support and its impact on job satisfaction and emotional exhaustion. Eur. J. Investig. Health Psychol. Educ. 13(12):2827–2840. doi:10.3390/ejihpe13120195 PMC10742909 38131894

[skaf400-B500] Green T. C. , Mellor D. J. 2011. Extending ideas about animal welfare assessment to include ‘quality of life’ and related concepts. N. Z. Vet. J. 59(6):263–271. doi:10.1080/00480169.2011.610283 22040330

[skaf400-B30] Greggor A. L. , Vicino G. A., Swaisgood R. R., Fidgett A., Brenner D., Kinney M. E., Farabaugh S., Masuda B., Lamberski N. 2018. Animal welfare in conservation breeding: Applications and challenges. Front. Vet. Sci. 5:323. doi:10.3389/fvets.2018.00323 30631770 PMC6315189

[skaf400-B31] Guilfoil K. 2022. Thousands of cattle dead amid continuing heat wave. ABC News. Available from https://abcnews.go.com/US/thousands-cattle-dead-amid-continuing-heat-wave/story? id=85434516. (Accessed May 29, 2025).

[skaf400-B32] Hackman J. R. , Oldham G. R. 1976. Motivation through the design of work: Test of a theory. Organ. Behav. Hum. Perform. 16(2):250–279. doi:10.1016/0030-5073(76)90016-7

[skaf400-B33] Hazel S. J. , Heberle N., McEwen M.-M., Adams K. 2013. Team-based learning increases active engagement and enhances development of teamwork and communication skills in a first-year course for veterinary and animal science undergraduates. J. Vet. Med. Educ. 40(4):333–341. doi:10.3138/jvme.0213-034R1 24077314

[skaf400-B34] Hemsworth P. H. , Coleman G. J. 2011. Human-livestock interactions: the stockperson and the productivity and welfare of intensively farmed animals. 2nd ed. Wallingford: CABI.

[skaf400-B35] Hobbs M. , Klachky E., Cooper M. 2020. Job satisfaction assessments of agricultural workers help employers improve the work environment and reduce turnover. Calif. Agr. 74(1):30–39.

[skaf400-B36] Jin M. H. , Guy M. E. 2009. How emotional labor influences worker pride, job satisfaction, and burnout: an examination of consumer complaint workers. Public Perform. Manage. Rev. 33(1):88–105. doi:10.2753/PMR1530-9576330104

[skaf400-B37] Jones J. S. , Murray S. R., Tapp S. R. 2018. Generational differences in the workplace. J. Bus. Divers. 18:88–97.

[skaf400-B38] Justen E. A. K. , Haynes C., VanDerZanden A. M., Grudens-Schuck N. 2009. Managers of Latino workers in the Iowa horticulture industry want educational programs to bridge language and cultural barriers. Horttechnology. 19(1):224–229. doi:10.21273/HORTTECH.19.1.224

[skaf400-B39] Kolstrup C. L. 2012. What factors attract and motivate dairy farm employees in their daily work? Work. 41 Suppl 1:5311–5316. doi:10.3233/WOR-2012-0049-5311 22317542

[skaf400-B40] Krogstad J. M. , Gonzalez-Barrera A. 2015. A majority of English-speaking Hispanics in the U.S. are bilingual. Pew Research Center. Available from: https://www.pewresearch.org/short-reads/2015/03/24/a-majority-of-english-speaking-hispanics-in-the-u-s-are-bilingual/ (Accessed May 29, 2025).

[skaf400-B41] Lacetera N. 2019. Impact of climate change on animal health and welfare. Anim. Front. 9(1):26–31. doi:10.1093/af/vfy032 32002236 PMC6951873

[skaf400-B42] Lewig K. A. , Dollard M. F. 2003. Emotional dissonance, emotional exhaustion and job satisfaction in call centre workers. Eur. J. Work Organ. Psychol. 12(4):366–392. doi:10.1080/13594320344000200

[skaf400-B43] Lyons S , Kuron L. 2014. Generational differences in the workplace: a review of the evidence and directions for future research: Generational differences in the workplace. J. Organ. Behav. 35(S1):S139–S157. doi:10.1002/job.1913

[skaf400-B44] Mills P. 2003. Group project work with undergraduate veterinary science students. Assess. Eval. Higher Educ. 28(5):527–538. doi:10.1080/02602930301676

[skaf400-B45] Moore I. C. , Coe J. B., Adams C. L., Conlon P. D., Sargeant J. M. 2014. The role of veterinary team effectiveness in job satisfaction and burnout in companion animal veterinary clinics. J. Am. Vet. Med. Assoc. 245(5):513–524. doi:10.2460/javma.245.5.513 25148093

[skaf400-B46] Muri K. , Tufte P. A., Coleman G., Moe R. O. 2020. Exploring work‐related characteristics as predictors of Norwegian sheep farmers’ affective job satisfaction. Sociol. Rural. 60(3):574–595. doi:10.1111/soru.12299

[skaf400-B47] Polachek A. J. , Wallace J. E. 2018. The paradox of compassionate work: a mixed-methods study of satisfying and fatiguing experiences of animal health care providers. Anxiety Stress Coping. 31(2):228–243. doi:10.1080/10615806.2017.1392224 29064289

[skaf400-B505] R Core Team . 2024. R: A language and environment for statistical ­computing. R Foundation for Statistical Computing, Vienna, Austria. https://www.R-project.org/

[skaf400-B48] Ramos A. K. , Carlo G., Grant K. M., Bendixsen C., Fuentes A., Gamboa R. 2018. A preliminary analysis of immigrant cattle feedyard worker perspectives on job-related safety training. Safety. 4(3):37. doi:10.3390/safety4030037

[skaf400-B49] Ramos A. K. , McGinley M., Carlo G. 2021a. Fatigue and the need for recovery among Latino/a immigrant cattle feedyard workers. J. Agromed. 26(1):47–58. doi:10.1080/1059924X.2020.1845894 33779518

[skaf400-B50] Ramos A. K. , McGinley M., Carlo G. 2021b. The relations of workplace safety, perceived occupational stress, and adjustment among Latino/a immigrant cattle feedyard workers in the United States. Saf. Sci. 139:105262. doi:10.1016/j.ssci.2021.105262

[skaf400-B51] Ramos A. K. , Reynaga D. 2023. The TN visa: the future of foreign workers in livestock production. J. Agromed. 28(1):81–85. 10.1080/1059924X.2022.2140734 36284467

[skaf400-B52] Ridge E. E. , Gill R., Daigle C. L. 2019. Evaluation of the Texas feed yard workforce: Survey of stockperson attitudes and perceptions towards euthanasia, animal care and employee value. Am. J. Anim. Vet. Sci. 14(2):139–150. doi:10.3844/ajavsp.2019.139.150

[skaf400-B53] Ritter C. , Russell E. R., Weary D. M., Von Keyserlingk M. A. G. 2021. Views of American animal and dairy science students on the future of dairy farms and public expectations for dairy cattle care: a focus group study. J. Dairy Sci. 104(7):7984–7995. doi:10.3168/jds.2020-19732 33896636

[skaf400-B54] Román-Muñiz I. 2023. Cultural awareness for veterinarians working with LatinX livestock caretakers. In: Proc. 6th Annu. AABP Recent Grad. Conf., Knoxville, TN, USA.

[skaf400-B55] Román-Muñiz IN. , Cramer M. C., Edwards-Callaway L. N., Stallones L., Kim E., Thompson S., Simpson H., Mijares S. 2021. Dairy caretaker perspectives on performing euthanasia as an essential component of their job. Animals. 11(2):289. doi:10.3390/ani11020289 33498843 PMC7912631

[skaf400-B56] Rosen E. 2010. Smashing silos. Bloomberg. Available from: https://www.bloomberg.com/news/articles/2010-02-05/smashing-silos (Accessed May 29, 2025).

[skaf400-B57] Rovai M. , Carroll H., Foos R., Erickson T., Garcia A. 2016. Dairy tool box talks: a comprehensive worker training in dairy farming. Front. Public Health. 4:136. doi:10.3389/fpubh.2016.00136 27471726 PMC4945628

[skaf400-B58] Sánchez E. , Gorgo-Gourovitch M., Stivers L. 2019. Creating a sense of belonging for Hispanic farmers and farmworkers in agricultural programming. Horttechnology. 29(4):476–481. doi:10.21273/HORTTECH04336-19

[skaf400-B59] Schullery N. M. 2013. Workplace engagement and generational differences in values. Bus. Commun. Q. 76(2):252–265. doi:10.1177/1080569913476543

[skaf400-B60] Scotney R. L. , McLaughlin D., Keates H. L. 2019. An investigation of the prevalence of compassion fatigue, compassion satisfaction and burnout in those working in animal-related occupations using the professional quality of life (ProQoL) scale. Vet. Nurse. 10(5):276–284. doi:10.12968/vetn.2019.10.5.276

[skaf400-B61] Silver J. K. , Ellinas E. H., Augustus-Wallace A. C. 2024. Sense of belonging is a critical component of workforce retention. BMJ. 384:q392. doi:10.1136/bmj.q392 38365283

[skaf400-B62] Sullivan P. A. , Varnum A., Bigler L., Cramer M. C., Román-Muñiz I. N., Edwards-Callaway L. N. 2025. Driving change: Exploring cattle transporters’ perspectives to improve worker and animal well-being. Transl. Anim. Sci. 9: txaf021. doi:10.1093/tas/txaf021 40083361 PMC11905222

[skaf400-B63] The Meat Institute. 2021. Lineamientos recomendados para el manejo animal y guía de auditorias. Available from: https://www.meatinstitute.org/sites/default/files/original%20documents/Animal_Handling_Guide_Spanish.pdf. (Accessed May 27, 2025).

[skaf400-B64] The National Dairy FARM Program. 2021. Cuidado apropiado para vacas no ambulatorias. Available from: https://nationaldairyfarm.com/wp-content/uploads/2018/10/DownCowPoster-Spanish.pdf (Accessed May 27, 2025).

[skaf400-B65] Thompson-Hughes J. 2019. Burnout and compassion fatigue within veterinary nursing: a literature review. Vet. Nurs. J. 34(10):266–268. doi:10.1080/17415349.2019.1646620

[skaf400-B66] Tsai H. S. , Shillair R., Cotten S. R., Winstead V., Yost E. 2015. Getting grandma online: Are tablets the answer for increasing digital inclusion for older adults in the U.S.? Educ. Gerontol. 41(10):695–709. doi:10.1080/03601277.2015.1048165 26877583 PMC4748942

[skaf400-B67] USDA ERS. 2023. United States Department of Agriculture Economic Research Service. U.S. employment in agriculture and support industries, 2001–23. Available from: https://ers.usda.gov/sites/default/files/_laserfiche/Charts/63451/QCEWEmployment2023_d.html? v=18517 (Accessed May 27, 2025).

[skaf400-B68] USDA NASS. 2025. United States Department of Agriculture National Agricultural Statistics Service. Cattle on feed. Available from: https://downloads.usda.library.cornell.edu/usda-esmis/files/m326m174z/w0894802v/x920hw28d/cofd0425.pdf (Accessed May 27, 2025).

[skaf400-B69] Valdivia C , Flores L. Y. 2012. Factors affecting the job satisfaction of Latino/a immigrants in the Midwest. J. Career Dev. 39(1):31–49. doi:10.1177/0894845310386478

[skaf400-B70] Vanhonacker F. , Verbeke W., Van Poucke E., Tuyttens F. A. M. 2008. Do citizens and farmers interpret the concept of farm animal welfare differently? Livest. Sci. 116(1–3):126–136. doi:10.1016/j.livsci.2007.09.006

[skaf400-B71] Wagner B. K. , Cramer M. C., Fowler H. N., Varnell H. L., Dietsch A. M., Proudfoot K. L., Shearer J., Correa M., Pairis-Garcia M. D. 2020. Determination of dairy cattle euthanasia criteria and analysis of barriers to humane euthanasia in the United States: Dairy producer surveys and focus groups. Animals. 10(5):770. doi:10.3390/ani10050770 32365463 PMC7278400

[skaf400-B72] Wallace J. E. 2019. Meaningful work and well‐being: a study of the positive side of veterinary work. Vet. Rec. 185(18):571–571. doi:10.1136/vr.105146 31563892

[skaf400-B73] Whitaker K. , Burnette A., Tan J., Graves M., Hunt J., Devine E., Anderson S., Kirkendall K., Wisnieski L. 2025. Factors influencing equine veterinarians’ job satisfaction and retention: a focus group study. Equine Vet. J. 57(6):1563–1571. doi:10.1111/evj.14467 39790082 PMC12508282

[skaf400-B74] Wilson D. J. , Parker E. M., Portillo-Gonzalez R., Ruegg P. L., Habing G. G. 2025. A focus group study exploring necessary competencies and contextual factors for effective antimicrobial stewardship on dairy farms. J. Dairy Sci. 108(5):5182–5192. 40043769 10.3168/jds.2024-25302 PMC12820904

[skaf400-B75] Winter-Collins A , McDaniel A. M. 2000. Sense of belonging and new graduate job satisfaction. J. Nurses Staff Dev. 16(3):103–111. doi:10.1097/00124645-200005000-00002 11913008

[skaf400-B76] Yang W , Renwick A. 2019. Consumer willingness to pay price premiums for credence attributes of livestock products–a meta‐analysis. J. Agric. Econ. 70(3):618–639. doi:10.1111/1477-9552.12323

